# Definitional ambiguity and the dual threat of Hypervirulent *Klebsiella pneumoniae* infections: a systematic review and meta-analysis

**DOI:** 10.1007/s15010-025-02708-4

**Published:** 2025-12-10

**Authors:** Danavath Nagendra, Veena Suresh, Ashritha A. Udupa, Thejesh Srinivas, Vandana Kalwaje Eshwara, Muralidhar Varma, Prabha Prakash, Shruthi Rao, Souvik Chaudhuri

**Affiliations:** 1 Department of Critical Care, Kasturba Medical College, Manipal, Manipal Academy of Higher Education, Manipal, Karnataka 576104 India; 2 Faculty of Science, Engineering and Built Environment, Deakin University, Burwood, VIC 3125 Australia; 3 Department of Microbiology, Kasturba Medical College, Manipal, Manipal Academy of Higher Education, Manipal, Karnataka 576104 India; 4 Department of Infectious Diseases, Kasturba Medical College, Manipal, Manipal Academy of Higher Education, Manipal, Karnataka 576104 India; 5 Department of Emergency Medicine, Kasturba Medical College, Manipal, Manipal Academy of Higher Education, Manipal, Karnataka 576104 India

**Keywords:** Liver abscess, Metastatic spread, Septic shock, Mortality, Carbapenem resistance

## Abstract

**Background:**

Hypervirulent *Klebsiella pneumoniae* (HvKp) causes severe invasive infections, but the lack of a standardized definition complicates surveillance. The emergence of carbapenem-resistant HvKp (CR-HvKp) poses a “dual-risk” threat whose impact is poorly quantified. This meta-analysis aimed to determine pooled proportions of severe outcomes stratified by diagnostic criteria and quantify the mortality risk associated with CR-HvKp.

**Methods:**

Following PRISMA guidelines, we systematically searched five databases for studies published up to July 2025 reporting clinical outcomes of HvKp infection. A random-effects model was used to calculate pooled proportions of liver abscess, metastatic spread, septic shock, microbiological failure and mortality, with subgroup analyses by HvKp definition (phenotypic, molecular, combined, or clinical). Odds ratios (OR) for mortality in CR-HvKp versus carbapenem-susceptible (CS)-HvKp were pooled.

**Results:**

From 4413 records, 79 studies involving 4240 patients were included. The pooled proportion of liver abscess was 24% (95% CI 17–32%) and metastatic spread was 22% (95% CI 12–32%), both significantly influenced by the diagnostic criteria used (p < 0.0001). Pooled mortality for HvKp was 21% (95% CI 15–27%). In stark contrast, pooled mortality for CR-HvKp was 57% (95% CI 35–78%). The frequency of microbiological failure among HvKp infected patients was reported to be 39%. A meta-analysis of six studies revealed CR-HvKp infection was associated with over 12-fold higher odds of death compared to CS-HvKp infection.

**Conclusion:**

HvKp continues to cause severe invasive infections, though outcome estimates vary due to inconsistent definitions. Mortality increases markedly when carbapenem resistance co-exists with virulence, indicating that poor outcomes are driven by both pathogenic and resistance mechanisms. Standardized diagnostic criteria and expanded genomic surveillance are essential to improve epidemiological comparability and guide early detection and containment of high-risk HvKp strains.

**Supplementary Information:**

The online version contains supplementary material available at 10.1007/s15010-025-02708-4.

## Introduction

The escalating global burden of antimicrobial resistance (AMR), compounded by the emergence of highly virulent pathogens, poses a critical threat to public health [1]. Among these, *Klebsiella pneumoniae* (Kp) is a major Gram-negative pathogen responsible for infections ranging from uncomplicated urinary tract infections to severe, rapidly progressive syndromes. Of particular concern is the hypervirulent *K. pneumoniae* (HvKp), first described in Taiwan in the 1980s, which is capable of causing invasive, community-acquired infections in otherwise healthy individuals [2].

**Fig. 1:**
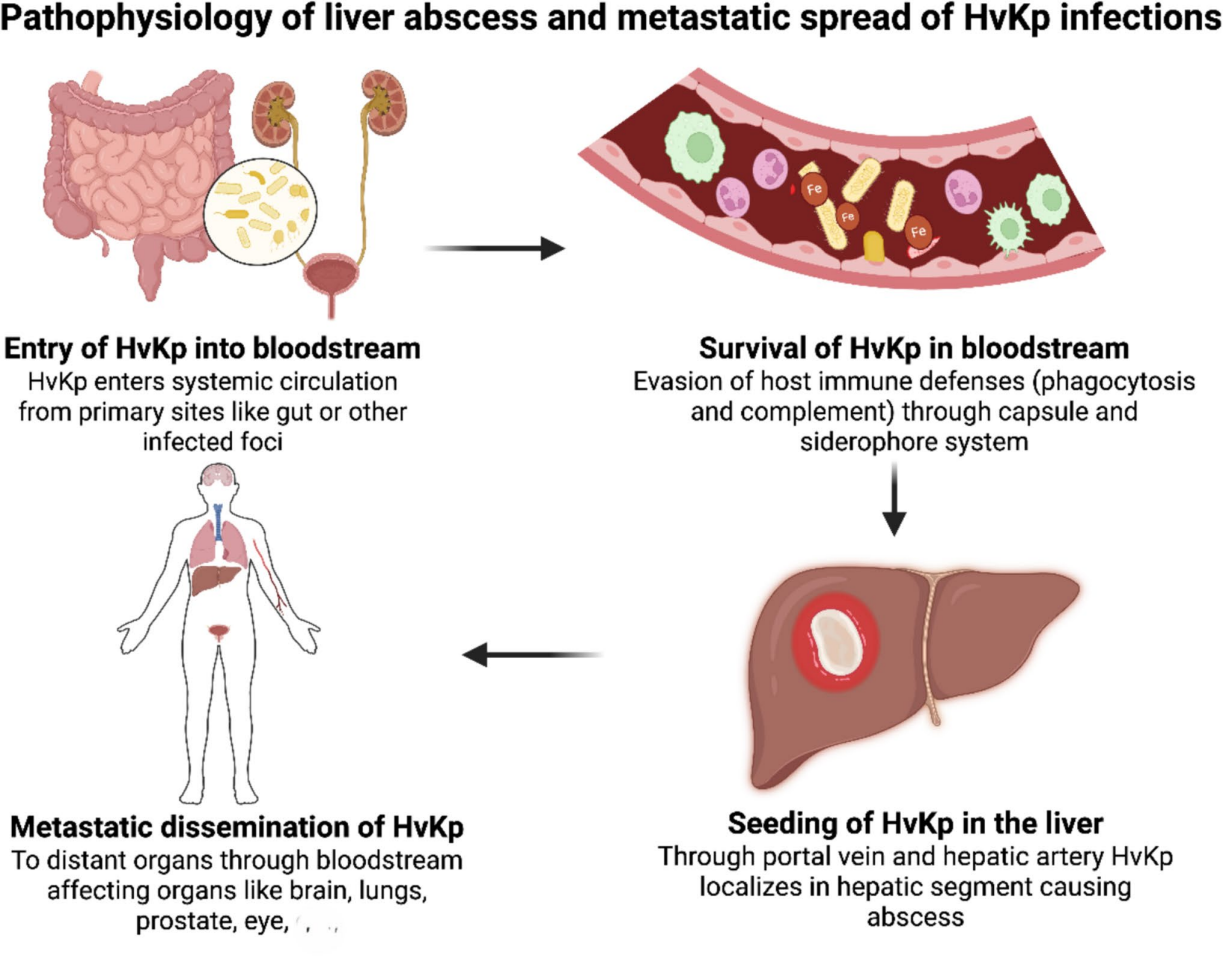
Graphical representation of the pathophysiology of liver abscess and metastatic spread in HvKp infection

HvKp is characteristically associated with pyogenic liver abscesses (PLA) and metastatic complications affecting the central nervous system (e.g., meningitis), eyes (e.g., endophthalmitis), and lungs. These invasive infections are driven by a pathogenic cascade in which the bacterium translocates across the gut barrier, seeds the liver, and disseminates systemically, often leading to septic shock and multiorgan failure (Fig. 1) [3–5]. However, whether this invasive clinical profile translates into a higher mortality risk remains debated. A prior meta-analysis suggested that despite its aggressive behaviour, HvKp infections do not consistently exhibit higher mortality than those caused by classical *K. pneumoniae* (cKp), likely due to HvKp’s historical susceptibility to antibiotics [4].

Although early HvKp isolates remained largely drug-susceptible, this therapeutic advantage is now eroding. Some HvKp strains have acquired carbapenem resistance (CR-HvKp), resulting in a dangerous convergence of hypervirulence and multidrug resistance [2, 6–8]. This “dual-risk” pathogen bridges two traditionally distinct niches, community-acquired virulence and hospital-acquired resistance creating a formidable clinical challenge [3].

A major obstacle to understanding HvKp’s epidemiology and outcomes is the absence of a universally accepted diagnostic definition [9, 10]. Studies have used diverse identification methods, such as the hypermucoviscosity (HMV) phenotype (string test), detection of virulence genes (e.g., *rmpA*, *iucA*, *iroB*, *peg-344*), or clinical syndromes (e.g., PLA), each with inherent limitations. The HMV phenotype lacks specificity, as some cKp strains are also HMV-positive, while some HvKp strains may not express HMV. Molecular markers offer higher specificity but are inconsistently applied, and gene panels vary between studies [4, 11–14].

Animal models such as mouse lethality assays provide high discriminatory power but are impractical for clinical use due to ethical and logistical constraints [15, 16]. Similarly, simpler models like *Galleria mellonella* larvae lack reliable specificity [17]. Defining HvKp based solely on clinical syndromes such as cryptogenic PLA [18, 19] also risks conflating disease presentation with pathogen identity. This definitional heterogeneity compromises epidemiological accuracy, complicates risk stratification, and hinders global surveillance efforts.

While a prior meta-analysis has quantified the burden of HvKp [4], none have stratified clinical outcomes according to the diagnostic definitions employed in primary studies. The extent to which methodological differences influence reported rates of liver abscess, metastatic spread, or mortality remains unclear.

In parallel, the emergence of CR-HvKp raises urgent clinical questions. How much more lethal is CR-HvKp compared to carbapenem-susceptible HvKp (CS-HvKp)? Do strains identified by specific diagnostic criteria correlate more strongly with severe outcomes? Clarifying these issues through structured evidence synthesis is essential for developing standardized diagnostic frameworks and guiding public health priorities.

Therefore, this study aims to assess the pooled proportions of key clinical outcomes including liver abscess, metastatic spread, septic shock, microbiological failure and mortality among patients with HvKp infections, stratified by diagnostic definition. The secondary objectives are to assess the overall mortality rate of CR-HvKp among all HvKp infected patients, and to evaluate mortality odds between CR-HvKp and CS-HvKp strains.

## Methodology

### Registration

This systematic review and meta-analysis was registered in PROSPERO (CRD420251116730) and conducted in accordance with PRISMA guidelines [20].

### Research question

What are the pooled proportions of key clinical outcomes, liver abscess, metastatic spread, septic shock, microbiological failure and mortality among HvKp infections, stratified by definition type (phenotypic, molecular, combined, or clinical presentation)? (Table 1).

**Table 1:** Research question, search strategy, and study inclusion criteria

PICOS	Description	Search string	Inclusion criteria	Exclusion criteria
Population	HvKp patients with specific definition	(Hypervirulent *Klebsiella pneumoniae* OR Hypermucoviscous *Klebsiella pneumoniae*)	Microbiologically diagnosed	Non-HvKp
Intervention/control	Not applicable			
Outcome	Liver abscess, metastatic spread, septic shock, microbiological failure, mortality		Liver abscess or metastatic spread or septic shock or microbiological failure or mortality reported including CR-HvKp	Studies with no outcome of interest
Studies			RCT, observational studies	Case reports, reviews, conference abstracts, letter to editor, correspondence, molecular studies, lab studies

*HvKp* Hypervirulent *Klebsiella pneumoniae*, *CR-HvKp* carbapenem resistant Hypervirulent *Klebsiella pneumoniae*, *RCT* randomised controlled trial

### Search strategy

We conducted a systematic bibliographic search across PubMed/MEDLINE, Embase, Scopus, Cochrane, and Web of Science. The search strategy combined keywords relevant to the definition of HvKp, with MeSH terms incorporated into the PubMed search (Table 1). Reference lists of the included studies were also examined to identify additional eligible publications.

### Article screening and selection

Following duplicate removal, two reviewers (DN and AU) independently screened the titles and abstracts of all retrieved articles. Full texts of potentially eligible studies were then assessed independently by the same reviewers, with any disagreements resolved by a third reviewer (SC). All studies published in English up to July 2025 were considered. The eligibility criteria are summarized in Table 1.

### Data extraction

Data from all patients with HvKp infection were extracted wherever available, without restriction to adults, to ensure capture of pooled proportions for key outcomes. Two reviewers independently collected publication details, study setting, time period of the study, total sample size (total *K. pneumoniae* cases and total HvKp cases), age of the HvKp group (where reported), the HvKp definition used in each study, diagnostic approach, complications including liver abscess, metastatic spread, and septic shock (number of events/total HvKp cases), antimicrobial resistance profile (carbapenem resistance, MDR], or extended-spectrum β-lactamase [ESBL] production in HvKp), and mortality (number of HvKp deaths/total HvKp cases).

For the primary analysis, studies were categorized based on the following explicit, a priori definitions:

- **Phenotypic:** Studies defining HvKp solely based on the string test, where a viscous string of > 5 mm was considered positive.
- **Molecular:** Studies defining HvKp based on the detection of one or more virulence-associated genes (e.g., *iucA*, *iroB*, *rmpA*, *rmpA2*, *peg-344 *etc.), specific sequence types, or capsular serotypes associated with hypervirulence.
- **Combined:** Studies requiring both a positive phenotypic test (string test) and the presence of at least one key molecular marker for HvKp classification.
- **Other (Clinical/Experimental):** Studies defining HvKp based on a characteristic clinical presentation (e.g., cryptogenic liver abscess) or the use of animal models (e.g., mouse lethality assay, *Galleria mellonella* infection model) in conjunction with molecular methods.

Microbiological failure was defined as the persistence of positive cultures beyond 3 days despite administration of adequate antimicrobial therapy [57]. The key clinical outcomes recorded were liver abscess, metastatic spread, septic shock, mortality and microbiological failure. All extracted data were entered into a Microsoft Excel spreadsheet.

### Risk of bias assessment

The risk of bias was evaluated using the Joanna Briggs Institute (JBI) critical appraisal tool for observational studies [21].

### Data synthesis

The pooled proportions of liver abscess, metastatic spread, septic shock, microbiological failure and mortality were calculated, with subgroup analyses performed based on the definition of HvKp for all outcomes. Sensitivity analyses were also conducted. Studies in which the primary diagnosis was pyogenic liver abscess were excluded from the pooled proportion of liver abscess unless virulence-specific data (i.e., number of strains positive for the hypermucoviscosity phenotype or aerobactin gene) were available. For mortality comparisons between CR-HvKp and CS-HvKp, a comparative meta-analysis was performed. The odds ratio (OR) for the risk of mortality in CR-HvKp versus CS-HvKp was computed from a 2 × 2 contingency table derived from the number of deaths and survivors in each group. Studies with zero events were retained with a cell value of zero following the Mantel–Haenszel odds ratio method. Pooled ORs and 95% confidence intervals were calculated using a random-effects model in Review Manager (RevMan) version 5.4.0. Pooled proportions were estimated using the inverse variance method with the Freeman–Tukey double arcsine transformation. Between-study heterogeneity was assessed using the Restricted Maximum Likelihood (REML) estimator, with Knapp–Hartung adjustment applied. Forest plots were generated using Meta-Analysis Online (https://metaanalysisonline.com/) [22]. Publication bias was evaluated through funnel plot visualization and both Begg’s rank correlation and Egger’s regression tests. Heterogeneity across studies was evaluated using the I^2^ statistic.

## Results

### Inclusion of studies

**Fig. 2:**
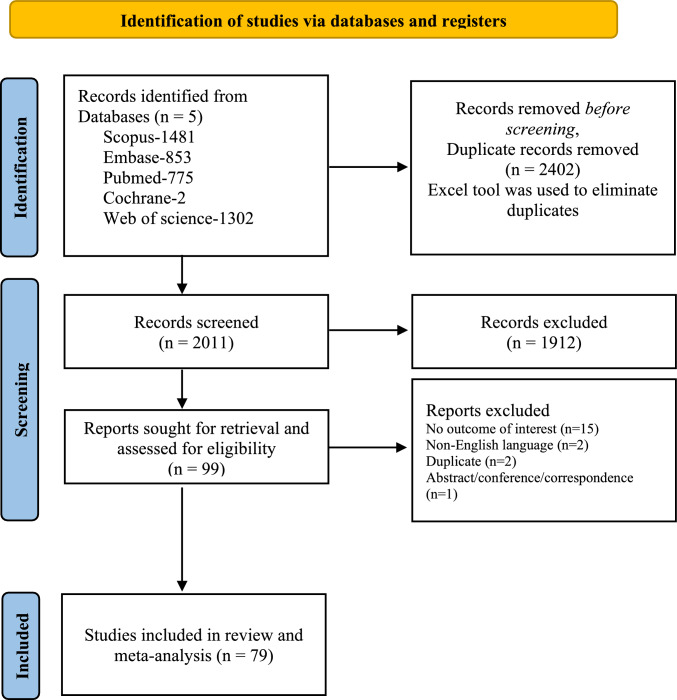
PRISMA flow chart. PRISMA flow diagram depicting the screening process and inclusion of studies involving virulent strains of *Klebsiella pneumoniae* that reported outcomes of interest. ‘Reports excluded’ indicates articles excluded after title & abstract screening or full-text review due to failure to meet eligibility criteria

A total of 4413 articles were retrieved from five databases. Following title-abstract and full-text screening, 79 studies met the eligibility criteria and were included in the final analysis. The PRISMA flow diagram (Fig. 2) has been updated to align with PRISMA 2020 recommendations, providing a breakdown of the reasons for exclusion at the full-text screening stage. Most studies originated from China and were retrospective in design. Across these studies, 4240 patients with HvKp were reported. The number of HvKp cases per study ranged from 4 to 232, with total *K*. *pneumoniae* cohort sizes ranging from 15 to 2002. Patient age in the HvKp group spanned 1–87 years, with the majority of studies reporting a mean or median age between 50 and 70 years. Geographically, approximately 65% of studies originated from China, followed by reports from Korea, Taiwan, Japan, and smaller numbers from India, France, Egypt, Malaysia, Indonesia, the USA, Argentina, and Canada.

Among the 79 included studies, 31 (39.24%) defined HvKp solely by phenotypic methods, 30 (37.97%) used exclusively molecular criteria, and 14 (17.72%) applied a combination of phenotypic and molecular definitions. Two studies (2.5%) identified HvKp based on clinical presentation, specifically cryptogenic liver abscess, while another two (2.5%) employed molecular methods in conjunction with animal models (mouse lethality assay or *Galleria mellonella* infection model). The clinical presentations studied included bloodstream infections/bacteremia, community-acquired and nosocomial infections, ventilator-associated pneumonia, pyogenic liver abscess, hepatobiliary infections, meningitis, and surgical site infections. Antimicrobial resistance patterns varied markedly: carbapenem resistance ranged from 0% in community-acquired infections to 100% in some hospital outbreaks of CR-HvKp. Multidrug resistance was reported in up to 87.5% of isolates, typically at higher rates among carbapenem-resistant strains refer Table 2.

**Table 2:** Clinical characteristics of the included studies

S. No.	Author ID (Year; country; study design & time period)	Total sample size (Total Kp; total HvKp)	Age of HvKp group	HvKp definition in the study	Diagnosis (main focus of the study)	Complications: liver abscess, metastatic spread, septic shock (No. events/total HvKp)	Resistance: carbapenem/MDR/ESBL in HvKp	Mortality (HvKp deaths/total HvKp)
1	Jung et al. (2013; Korea; Retrospective multi-centre (June–Aug 2010)) [29]	Total Kp: 33; HvKp: 14 (42.4%)	Not reported	Hypermucoviscous (HV) isolates defined by string test (> 5 mm)	Bacteraemia	Liver abscess: 3/14 (21.4%); Metastatic spread: 11/14 (78.6%); Septic shock: not specified	Carbapenem: none (0%) resistant to imipenem or meropenem MDR: lower resistance rates than non-HV strains ESBL: none (0%) produced ESBLs	4/14 (28.6%)
2	Li et al.; 2014; China; Retrospective single centre (Apr 2010–Jun 2012) [2]	Total Kp: 88; HvKp: 29 (33%)	Mean age 51.4 ± 12.1 years (for all); HvKp-specific not stated (not reported)	HvKP isolates defined as string test positive (> 5 mm)	NA	Liver abscess: 7/29 (24%); Metastatic spread: 18/29 (62%); Septic shock: not explicitly mentioned	Carbapenem: none (0%) resistant MDR: 15.4–25.0% resistant to ≥ 3 antimicrobials (excluding ampicillin) ESBL: 5/29 (17%)	4/29 (13.8%)
3	Liu et al. (2014; Mainland China; Retrospective cohort study (June 2008–April 2012)) [33]	Total Kp: 70; HvKp: 22 (31.4%)	9/22 (40.9%) were > 60 years	HvKP defined by string test positive (> 5 mm)	Bacteremia	Liver abscess: 4/22 (18.2%); Metastatic spread: 2/22 (9.1%); Septic shock: not explicitly mentioned	Carbapenem: 0/22 (0%); MDR: not explicitly reported ESBL: 2/22 (9.09%)	1/17 (5.88%) 5 (no follow-up)
4	Qu et al. (2015; East China; Retrospective review (June 1, 2008, to June 30, 2012)) [12]	Total Kp: 45; HvKp: 13 (28.9%)	52.3 ± 8.7 years (mean ± SD)	Hypermucoviscosity phenotype defined by string test (> 5 mm)	*K. pneumoniae* liver abscess	Liver abscess: 13/13 (100%, all patients had KLA) Metastatic spread: 5/13 (38.5%); Septic shock: 2/13 (15.4%)	Carbapenem: all (100%) susceptible to imipenem and meropenem MDR: not explicitly reported ESBL: 1/13 (7.7%)	0/13 (0%)
5	Yan et al. (2016; China; Retrospective study (January 2014–December 2014)) [13]	Total Kp: 49; HvKp: 14 (28.6%)	Median 55.5 (42.8–62.8) years	Strains possessed p-rmpA and iroB and iucA were defined as hvKP	Ventilator-associated pneumonia	Liver abscess: Not directly studied as a complication in this cohort Metastatic spread: 0/14 (0%); Septic shock: not explicitly mentioned	Carbapenem: 0/14 (0%) resistant to Imipenem and Ertapenem MDR: not explicitly reported ESBL: 1/14 (7.1%)	0/14 (0%)
6	Yu et al. (2016; Taiwan; Retrospective study (January 2009 through June 2010)) [24]	Total Kp: 48; HvKp: 19 (39.6%)	Median 68(57–76) years	Isolates in the hypervirulence group included any of the following virulence determinants: hypermucoviscosity phenotype, rmpA gene, or rmpA2 gene. All isolates used were non-K1/K2 strains	Bacteremia caused by extended-spectrum β-lactamase-producing *Klebsiella pneumoniae*	Liver abscess: NA; Metastatic spread: NA Septic shock: NA	Carbapenem: NA MDR: NA ESBL: All 19 HvKp isolates were ESBL-producing	10/19 (52.6%)
7	Zhang et al. (2016; China; Nationwide multicentre prospective surveillance (February–July 2013)) [14]	Total Kp: 230; HvKp: 87 (37.8%)	Mean 55.9 ± 1.5 years	HvKP was defined by aerobactin detection using PCR	NA	Liver abscess: 10/87 (11.5%); Metastatic spread: 11/87 (12.6%); Septic shock: 6/87 (6.9%)	Carbapenem: one ESBL-producing HvKp isolate resistant to carbapenem MDR: not explicitly reported ESBL: 11/87 (12.6%)	2/71 (2.8%)
8	Wu et al. (2017; China; Retrospective study (October 2010–December 2014)) [25]	Total Kp: 165; HvKp: 64 (38.8%)	Not explicitly stated for HvKp group	HvKP strains were defined as those that were positive for at least two of the following three indicators: hypermucoviscosity of colony (string test positive), gene amplification for rmpA, and amplification of aerobactin	Bacteremia and at least one other body site infection	Liver abscess: 14/27 (51.9%); Metastatic spread: 2/27 (7.4%); Septic shock: NA	Carbapenem: 1/28 (3.6%) KPC-2 gene positive MDR: not explicitly reported ESBL: 5/28 (17.9%)	NA
9	Guo et al. (2017; China; Prospective study (January 2013 to October 2015)) [11]	Total Kp: 369; HvKp: 84 (22.8%)	≤ 60 years old: 39 (46.4%); > 60 years old: 45 (53.6%); Male patients with 41–50 years old: 13 (15.5%)	Hypermucoviscosity phenotype determined by string test (> 5 mm)	Invasive infections	Liver abscess: 38/84 (45.2%); Metastatic spread: NA; Septic shock: NA	Carbapenem: 4/84 (4.8%) MDR: 3/84 (3.57%) ESBL: NA	NA
10	Jiayang Li et al. (2018; China; Retrospective study (September 2015–December 2016)) [34]	Total Kp: 143; HvKp: 35 (24.5%)	Mean age was 54.9 ± 17.1 years	A positive polymerase chain reaction (PCR) amplification of the plasmid-borne rmpA (p-rmpA) and aerobactin (iucA) was identified as hvKP	Bloodstream infections	Liver abscess: 5/35 (14.3%); Metastatic spread: NA; Septic shock: 18/35 (51.4%)	Carbapenem: 20/35 (57.1%) KPC-producing Imipenem 19/35 (54.3%), meropenem 20/35 (57.1%); MDR: NA; ESBL: NA	13/35 (37.1%)
11	Xu et al. (2018; China; Retrospective cohort study (January 2013–December 2015)) [35]	Total Kp: 285; HvKp: 69 (24.2%)	Mean age for blaKPC−/HM+ subgroup was 57.1 ± 13.9 years blaKPC+/HM+ subgroup was 68.0 ± 19.2	Hypermucoviscosity phenotype determined by string test (> 5 mm)	Bloodstream infections	Liver abscess: 15 (22.7%) in blaKPC−/HM+ subgroup, 0 in blaKPC+/HM+ subgroup; Metastatic spread: NA; Septic shock: 10 (15.2%) in blaKPC−/HM+ subgroup Septic shock: 2 (66.7%) in blaKPC+/HM+ subgroup	Carbapenem: 3 HMKP isolates harboured blaKPC​ (carbapenemase gene); MDR: NA; ESBL: NA	blaKPC−/HM+ subgroup: 5/66 (7.6%); blaKPC+/HM+ subgroup: 3/3 (100%)
12	Liu et al. (2018; China; Retrospective study (June 2008–July 2017)) [36]	Total Kp: 202; HvKp: 96 (47.5%)	Mean age was 83.24 ± 7.35 years	HvKp was defined as aerobactin positive	NA	Liver abscess: 10/96 (10.4%); Metastatic spread: NA; Septic shock: 16/96 (16.7%)	Carbapenem: 12/96 (12.5%) CR-HvKP strains; MDR: 24/96 (25.0%); ESBL: 25/96 (26.0%)	16/96 (16.7%)
13	Liu and Guo (2018; China; Retrospective study (November 2008–December 2017)) [37]	Total Kp: 73; HvKp: 34 (46.6%)	Mean age was 83.06 ± 8.55 years	HvKp is defined as aerobactin positive	Ventilator-associated pneumonia	Liver abscess: NA; Metastatic spread: NA; Septic shock: 16/34 (47.1%)	Carbapenem: 8 CR-HvKp isolates; MDR: 14/34 (41.2%); ESBL: 13/34 (38.2%)	14/34 (41.2%)
14	Rasha El-Mahdy et al. (2018; Egypt; Cross-sectional study (June–December 2015)) [38]	Total Kp: 65; HvKp: 4 (6.2%)	2/4 (> 60 years old), no mean age provided	HvKP was identified by the presence of either the iucA or iroB gene	Hospital-acquired Infections	Liver abscess: NA; Metastatic spread: NA; Septic shock: NA	Carbapenem: All hvKP isolates sensitive to carbapenem; MDR: all hvKP strains were MDR; ESBL: 1 hvKP isolate was an ESBL producer	2/4 (50%)
15	Liu and Guo (2019; China; Retrospective study (January 2008–January 2014)) [32]	Total Kp: 175; HvKp: 80 (45.7%)	Mean age was 83.2 ± 8.75 years	Hypermucoviscous and aerobactin positive	NA	Liver abscess: 8/80 (10.0%); Metastatic spread: 23/80 (28.8%); Other abscesses: 13/80 (16.3%); Septic shock: 9/80 (11.3%)	Carbapenem: 2 HvKp isolates resistant to carbapenems; MDR: 16/80 (20.0%); ESBL: 13/80 (16.3%)	14/80 (17.5%)
16	Xu et al. (2019; China; Retrospective cohort study (January 2011–July 2017)) [39]	Total Kp: 48 (44 available isolates); HvKp: 22 (45.8%)	Mean age was 55.2 ± 13.4 years	HvKP was defined as the presence of pLVPK-like virulence plasmid	Meningitis	Liver abscess: 1/22 (4.5%); Metastatic spread: NA; Septic shock: NA Bacteremia: 9/22 (40.9%)	Carbapenem: 15/22 (68.2%); KPC-2: 15/22 (68.2%); ESBL: 13/22 (59.1%) for CTX-M-9 group, 3/22 (13.6%) for CTX-M-1 group, 10/22 (45.5%) for SHV genes	17/22 (77.3%) for hvKP group; 15/15 (100%) for KPC-2-producing hvKP
17	Namikawa et al. (2019; Japan; Retrospective review (January 2012–April 2018)) [40]	Total Kp: 114; HvKp: 24 (21.1%)	Mean age of 67.8 years	Hypermucoviscosity phenotype determined by string test (> 5 mm)	Bacteremia	Liver abscess: 3/24 (13%); Metastatic spread: NA; Septic shock: NA	Carbapenem: 0/24 (0%) resistant to imipenem; MDR: NA; ESBL: 0/24 (0%)	7/24 (29.2%)
18	Zhao et al. (2020; China; Research article (September 2008 and July 2017)) [41]	Total Kp: 51; HvKp: 26 (51%)	Mean age was 46.0 ± 16.7 years	HvKp was defined based on the presence of at least two of the following indicators: a positive string test, rmpA/rmpA2 gene-positive status, and/or aerobactin-positive status	Surgical site infections	Liver abscess: NA; Metastatic spread: NA; Septic shock: NA	Carbapenem: 2/26 (7.7%); MDR: NA; ESBL: 4/26 (15.4%)	0/26 (0.0%)
19	Hwang et al. (2020; Korea; Retrospective study (January 2014 and December 2014)) [42]	Total Kp: 91; HvKp: 39 (42.9%)	NA	Strains that possessed rmpA and iutA were defined as hvKP	Pneumonia	Liver abscess: NA; Metastatic spread: NA; Septic shock: NA	Carbapenem: NA; MDR: NA; ESBL: NA	7/39 (17.9%)
20	Harada et al. (2019; Japan; Cross-sectional study (December 2013 through March 2014)) [43]	Total Kp: 140; HvKp: 26 (18.6%)	Median age of 75.5 years (IQR 68.5–83.5)	HvKp was defined as *K*. *pneumoniae* isolates carrying any of the virulence genes: rmpA, rmpA2, iroBCDN	Bacteremia	Liver abscess: 4/26 (15.4%); Metastatic spread: 3/26 (11.5%); Septic shock: 6/26 (23.1%)	Carbapenem: 0/26 (0%) carbapenemase gene; MDR: not explicitly reported; ESBL: 1/26 (3.8%) carried blaCTX-M-2	2/26 (7.7%)
21	Liu et al. (2020; China; Retrospective study (2008–2018)) [44]	Total Kp: 158; HvKp: 79 (50%)	Mean age of total cohort 78.18 ± 15.16 years Community-acquired infection HvKP: 75.29 ± 9.12; Healthcare-associated infection: 76.84 ± 17.95; Nosocomial infection: HvKp:78.21 ± 18.09	Defined by the presence of some combination of prmpA, prmpA2, iucA, iroB, and peg-344, genes shown to accurately identify HvKp	NA	Liver abscess: 2/79 (2.5%); Metastatic spread: 31/79 (39.2%); Septic shock: NA	Carbapenem: 16/79 (20.3%), (Healthcare associated HvKp infections: 2/19 (11%) and > cKp: 1/18 (5.6%), Nosocomial infection: 14/53 (26.4%)); MDR: 31/79 (39.2%), (Healthcare associated HvKp infections: 4/19 (21.6%), Nosocomial infection: 27/53 (51%)); ESBL: 31/79 (39.2%), (Community-acquired infection: 1/7 (14.3%), Healthcare associated HvKp infections: 5/31 (16.1%), Nosocomial infection: 25/53 (47.2%))	10/79 (12.7%)
22	Su et al. (2021; China; Retrospective analysis (July 2019–March 2020)) [45]	Total Kp: 115; HvKp: 68 (59.1%)	Mean age was 57.01 ± 19.00 years	When rmpA, rmpA2, and string test were all positive, the strain is defined as HvKP	NA	Liver abscess: 8/68 (11.8%); Metastatic spread: NA; Septic shock: NA	Carbapenem: 12/68 (17.6%) were carbapenem resistant; MDR: not explicitly reported; ESBL: not explicitly reported	NA
23	Ding et al. (2022; China; Retrospective study (October 2016–November 2018)) [46]	Total Kp: 123; HvKp: 53 (43.1%)	Mean age of HvKp cohort 53.6 ± 16.5 years	An isolate that positive for both PCR amplification of aerobactin gene and Galleria mellonella infection model was defined as HvKp	NA	Liver abscess: 37.7%; Metastatic spread: 54.7%; Septic shock: NA	Carbapenem: 1/53 (1.9%) resistant to imipenem, ertapenem, meropenem; MDR: 15.1%; ESBL: 7.5%	NA
24	Yang et al. (2022; China; Retrospective study (January–December 2019)) [30]	Total Kp: 88; HvKp: 69 (78%)	Mean age was 60.84 ± 12.36 years	The hvKP strains in this study were considered by the presence of the peg-344 or iucA gene	*Klebsiella pneumoniae* infection of the hepatobiliary system	Liver abscess: 61/69 (88.4%); Metastatic spread: NA; Septic shock: NA	Carbapenem: 1/69 (1.4%); MDR: NA; ESBL: NA	3/69 (4.3%)
25	Sheng et al. (2022; China; Retrospective analysis (January 2019–March 2020)) [31]	Total Kp: 66; HvKp: 29 (43.9%)	Median age 58 (50–73) years	HvKP was defined as aerobactin positive	Bacteremia	Liver abscess: 1/22 (4.5%); Metastatic spread: NA; Septic shock: NA	Carbapenem: none of the hvKP strains were carbapenem resistant; MDR: NA; ESBL: NA	4/29 (13.8%)
26	Vandhana et al. (2022; India; Prospective study (January 2021–May 2021)) [3]	Total Kp: 129; HvKp: 18 (13.9%)	NA	Aerobactin positive strains were designated as Hv-Kp	NA	Liver abscess: NA; Metastatic spread: NA; Septic shock: NA	Carbapenem: NA; MDR: 44.44%; ESBL: 44.44%	16/18 (88.9%)
27	Raj et al. (2022; India; Retrospective study (2 years, ending August 2022)) [47]	Total Kp: 120; HvKp: 14 (11.6%)	Predominantly belonged to age groups 19–30 years and 31–55 years	HvKp was phenotypically identified by string test and genotypically confirmed by the presence of the iucA gene using PCR	NA	Liver abscess: NA; Metastatic spread: NA; Septic shock: NA	Carbapenem: 78% resistant to carbapenems; Healthcare-associated infections-9/14 (64.3%); MDR: 87.5%; ESBL: 92% resistant to third-generation cephalosporins Healthcare-associated infections-12/14 (86%)	4/14 (28%)
28	Huang et al. (2023; China; Retrospective study (January 2020–January 2021)) [7]	Total Kp: 109; HvKp: 45 (41.3%); CR-HvKp: 24/45 (53.3%)	HvKp cohort: Median age 60 (51.0–68.0) years; CR-HvKp cohort: Median age 60.0 (54.0–69.8)years	HvKP was identified as combinations of genes rmpA, rmpA2, iucA, iroB or peg-344	NA	HvKp: Liver abscess: 4/45 (8.9%); Metastatic infection: 14/45 (31.1%); Septic shock: NA CR-HvKp cohort: Liver abscess: 0 Metastatic infection: 9 (37.5%); Septic shock: NA	Carbapenem: 24/45 (53.3%) expressed carbapenemases; 8 NDM-1-KPC-2-CR-hvKP strains identified; MDR: not explicitly reported; ESBL: Not explicitly reported	HvKp: 8/45 (17.8%) CR-HvKp-8/24 (33.3%)
29	Kim et al. (2023; Korea; Retrospective cohort study (March 2018–December 2019)) [27]	Total Kp: 179; HvKp: 67 (37.4%)	Mean age 70.56 ± 12.11 years	Hypermucoviscosity was defined by the string test	Bacteremia from non-hepatobiliary tract infection	Liver abscess: not directly studied as primary focus was non-hepatobiliary; Metastatic infection: 6/67 (9%); Septic shock: 32/67 (48%)	Carbapenem: NA; MDR: NA; ESBL: NA	28/67 (42%)
30	Yang et al. (2023; China; Retrospective study (2018 and 2021)) [48]	Total Kp: 54; HvKp: 33 (61.11%)	Median age of 60.5 years (25th and 75th percentiles, 24–87 years)	HvKp was identified by checking for the presence of four of five genes (iroB, iucA, rmpA, rmpA2, and peg-344)	Pyogenic infections	Liver abscess: 15/33 (45.45%); Metastatic spread: NA; Septic shock: 7/33 (21.21%)	Carbapenem resistance: NA; MDR: NA; ESBL: not explicitly mentioned	6/33 (18.18%) had poor prognosis
31	Rafat et al. (2018; France; Prospective monocentric observational study (September 2011–November 2016)) [49]	Total Kp: 59 infections; HvKp: 12 HvKp infections (20.3%)	Median age of 55.2 (44.5–59.3) years	*K*. *pneumoniae* strains were screened for hypermucoviscosity based on a string test	NA	Liver abscess: 2/12 (17%); Metastatic spread: 3/12 (25%); Septic shock: 8/12 (67%)	Carbapenem: NA; MDR: NA; ESBL: NA	6/12 (50%)
32	Cubero et al. (2016; Spain; Retrospective study (2007–2013)) [50]	Total Kp: 878; HvKp: 53 (6%) hypermucoviscous isolates	magA + /rmpA + 56.7 ± 8.3 ***magA***^**−**^**/*rmpA***^**−**^ 69.3 ± 15.6	Hypermucoviscosity was defined by the string test	Bacteremia	Liver abscess: 8/53 (16%); Metastatic spread: NA; Septic shock: 13/53 (25%)	NA	9/53 (17%)
33	Guo et al.; 2016; mainland China; Retrospective (Jan 2012—Aug 2014) [51]	Total Kp: 70; Total HvKp: 14 (20%)	64 ± 14	The formation of a viscous string of > 5 mm confirmed the HV-positive phenotype	Ventilator-associated pneumonia	Liver abscess: None/14 (0%); Metastatic spread: None/14 (0%); Septic shock: not specified	Carbapenem: 0/14 (0%); MDR: not specified; ESBL: 2/14 (14.3%)	8/14 (57.1%)
34	Hao et al.; 2019; Wenzhou, China; Observational, cross-sectional (Jan—Oct 2016) [52]	Total Kp: 48; Total HvKp: 33 (68.8%)	55.48 ± 13.5 years (mean ± SD)	Hypermucoviscosity was defined by the string test	Community-acquired *K. pneumoniae* bloodstream infections	Liver abscess: 11/33 (33.3%); Metastatic spread: Not specified; Septic shock: Not specified	Carbapenem: 1/33 (3.0%) resistant to imipenem; MDR: only one of the seven multidrug-resistant clinical isolates was an hvKP isolate; ESBL: 2/33 (6.1%)	Not reported
35	Chen et al.; 2022; China; Retrospective (Jan 2015–Jan 2019) [53]	Total Kp: 225; Total HvKp: 114 (50.67%)	63 (55, 73) years (median, IQR)	Hypermucoviscosity was defined by the string test	Severe infection in surgical intensive care unit (ICU)	Liver abscess: 79/114 (69.3%) (primary infection site); Metastatic spread: Not specified; Septic shock: 19/114 (16.7%)	Carbapenem: not specified; MDR: not specified; ESBL: not specified	16/114 (14.0%) (28-day mortality rate)
36	Yang et al.; 2020; North China; Original Article (isolates collected in 2015) [54]	Total Kp: 113; Total HvKp: 59 (52.21%)	65.64 ± 1.805 years (mean ± SD)	HvKp strains were defined by PCR amplification of virulence related genes: plasmid-borne rmpA (p-rmpA, p-rmpA2)	NA	Liver abscess: 2/59 (3.39%); Metastatic spread: Not specified; Septic shock: Not specified	Carbapenem: NA; MDR: not specified; ESBL: 1/59 (1.69%)	Not reported
37	Lee et al.; 2006; Taiwan; Retrospective observational study (June 1999–June 2001) [55]	Total Kp: 308 (bacteraemic episodes); Total HvKp: 99 (32%) (from all Kp, 83 from CA-KpB, 16 from HA-KpB)	9.3 ± 12.8 years (mean ± SD) (for HV-positive CA-KpB)	Hypermucoviscosity was defined by the string test	Community-acquired *K. pneumoniae* bacteraemia	Liver abscess: 27/83 (32.5%); Metastatic spread: 31/83 (37.3%); Septic shock: not specified	Carbapenem: not specified; MDR: not specified; ESBL: not specified	48 h: 13/83 (16.5%); 14 days: 21/83 (26.9%)
38	Peirano et al.; 2013; Calgary, Alberta, Canada; retrospective cohort (Jan 2001–Dec 2007) [56]	Total Kp: 134; Total HvKp: 10 (8.2%)	63 years (mean)	Hypermucoviscosity was defined by the string test	Community-acquired bacteremia	Liver abscess: 4/10 (40%); Metastatic spread: NA; Septic shock: Not specified	Carbapenem: not specified; MDR: not specified; ESBL: not specified	Case fatality rate: 10% (1/10)
39	Fauvet et al.; 2020; New Caledonia; Observational retrospective study (May 2013–March 2015) [57]	Total Kp: 55; Total HvKp: 15 (27%)	55.7 (± 11.7) years (mean ± SD)	Hypermucoviscosity was defined by the string test	Bacteremia	Liver abscess: 2/15 (13.3%); Metastatic spread: NA; Septic shock: not specified	Carbapenem: not specified; MDR: not specified; ESBL: 1/15 (6.7%)	Infection-related death: 7/15 (46.7%)
40	Zhuo et al.; 2025; Beijing, China; Retrospective study (Jan 1, 2022–June 20, 2023) [16]	Total Kp: 287; Total HvKp: 70 (24.4%)	59 years (59 ± 16) (mean ± SD)	K1, K2, and K20 *K. pneumoniae* were defined as hvKp	Lower respiratory tract infections	Liver abscess: 6/70 (8.6%); Metastatic spread: 3/70 (4.3%) (Others); Septic shock: 10/70 (14.3%)	Carbapenem: carbapenemase gene blaKPC−2 (0%); MDR: not explicitly quantified; ESBL: ESBL genes blaCTX−M−14 (0%), blaCTX−M−15 (1.4%), blaCTX−M−65 (0%)	In-hospital mortality: 6/70 (8.6%)
41	Tang et al.; 2025; Beijing, China; Longitudinal cohort study, systematic retrospective (2017–2023) [58]	Total Kp: 1179; Total HvKp-p: 127 (10.7%)	63.3 ± 18.0 years (mean ± SD)	The presence of all five of the virulence-associated genes: iucA, iroB, peg-344, rmpA, and rmpA2	NA	Liver abscess: not specified; Metastatic spread: not specified; Septic shock: 29/126 (23.0%)	Carbapenem: none of the hvKp-p isolates possessed carbapenemases; MDR: significantly lower AMR gene carriage (0.2 ± 0.9); ESBL: 2/127 (1.6%)	14-day mortality: 18/106 (17.0%); 28-day mortality: 20/106 (18.9%); 90-day mortality: 21/106 (19.8%)
42	Yu et al.; 2006; Taiwan; Retrospective (July 2003–Dec 2004) [23]	Total Kp: 151 (from bacteremia cases); Total HvKp: 58 (38%) (hypermucoviscosity prevalence)	NA	Strains were defined as hypermucoviscosity positive when the viscous strings were > 5 mm	NA	Liver abscess: 13/58 (22.4%) (of HMV-positive isolates); Metastatic spread: not specified; Septic shock: not specified	Carbapenem: not specified; MDR: not specified; ESBL: not specified	Not specified
43	Liu et al.; 2025; China; Retrospective study (2014–2018) [8]	Total Kp: 2,002 Klebsiella pneumoniae isolates; Total HvKp: 127 (6.3%) CR-HvKp:9/127 (7.1%); Hv-CRKp:116/127 (91.3%)	Age distribution for hv-CRKP (all deaths in this group): 0–17 years: 0 (0.0%); 18–49 years: 10 (40.0%); 50–64 years: 6 (24.0%); 65 + years: 9 (36.0%)	HvKp strains were identified by the presence of virulence genes (rmpA, rmpA2, iroB, iucA)	NA	Liver abscess: NA; Metastatic spread: NA; Septic shock: 32/125 (26%) (in hv-CRKP)	Carbapenem: All 127 CR-HvKp. Genes: *blaKPC-2* (122/127) (96%), *blaNDM-1* (7/127) (5.5%) MDR: NA ESBL: NA	25/125 (20%) (in hv-CRKP)
44	Chen et al.; 2025; Western China; Retrospective study (January–December 2024) [59]	Total CRKP isolates: 68; Total CR-HvKP isolates: 36 (52.9%)	Median age of 62 years (interquartile range: 45, 73)	CR-HvKP was defined as the presence of any one of rmpA, rmpA2, iroB, iucA, and peg-344	NA	Liver abscess: 2/32 (6.3%); Metastatic spread: NA; Septic shock: NA	Carbapenem: All 36 CR-HvKP. Genes: *blaKPC-2* (91.7% of CR-HvKP) MDR: NA ESBL: NA	5/23 (22%) 9 got transferred
45	Sun et al. (2025; China; Retrospective analysis from January 2019 to November 2024) [60]	Total Kp: 381; HvKp (hmKp): 232 (61%)	62 years (median)	String test > 5 mm	Community-acquired *Klebsiella pneumoniae* infections	Liver abscess: Not reported; Metastatic spread: 67/232 (28.9%); Septic shock: 90/232 (38.8%)	Carbapenem (Imipenem): 3%; MDR: 6.5%; ESBL: 6.5%	71/232 (30.6%)
46	Nannini et al. (2024; Argentina; Retrospective chart review between October 2015 and November 2018) [61]	Total patients: 15; HvKp isolates studied for molecular analysis: 8 (53.3%)	60 years (mean)	Combination of both phenotype and genotype positive strains	Cryptogenic liver abscess	Liver abscess: 15/15 (100%); Metastatic spread: 11/15 (73%); Septic shock: Not specified	Carbapenem: 0% (all isolates susceptible to tested antibiotics except ampicillin); MDR: NA; ESBL: NA	0/15 (0%) (No in-hospital deaths)
47	Moutel et al. (2024; France; Monocentric retrospective study from January 2016 to December 2020) [62]	Total Kp BSI: 70; HvKp: 9 (13%)	50 years (41–68) (median IQR)	Defined by detection of both rmpA and iutA	Bacteremia	Liver abscess: Not specified; Metastatic spread: Not specified; Septic shock: 4/9 (44%)	Carbapenem: NA; MDR: NA; ESBL: NA	4/9 (44%) (30-day mortality)
48	Hyun et al. (2024; South Korea; Retrospective analysis between December 2013 and November 2015) [63]	Total Kp: 414; HvKp (CLA-defined): 34 (8.2%)	66.6 ± 10.9 years (mean ± SD)	In this study, we defined hypervirulent *K*. *pneumoniae* as CLA from a clinical perspective String test was positive for 25 out of 34 CLA Aerobactin was positive for 27 out of 34 CLA	Community-acquired liver abscess	Liver abscess: 34/34 (100%); Metastatic spread: 1/34 (2.9%); Septic shock: 9/34 (26.5%)	Carbapenem (Ertapenem): 0%; Carbapenem (Imipenem): 0%; MDR: NA; ESBL: 1/34 (2.9%)	2/34 (5.88)
49	Huang et al. (2023; China; Bicentric retrospective study from September 2017 to September 2022) [64]	Total HvKp: 116	55.94 ± 15.93 years	KP-infected patients with string test positive. KP strains with one or more of genotype (rmpA, rmpA2, iucA, iroB, magA and peg344) positive	NA	Liver abscess: 12/116 (10.3%); Metastatic spread: NA; Septic shock: 47/116 (40.5%)	Carbapenem: NA; MDR: NA; ESBL: NA	33/116 (28.4%) (30-day all-cause mortality)
50	Guo et al. (2023; China; Retrospective study from January to December 2020) [9]	Total patients: 266 (129 VAP, 137 PLA); HvKp (PLA group): 25 (18.2%); HvKp (VAP group): 14 (11%)	PLA group: 53.31 ± 17.54 years; VAP group: 67.86 ± 23.13	The combination of *iucA*, *rmpA*, hypermucoviscous phenotype, and ST23 presented in *K. pneumoniae* infection	Ventilator-associated pneumonia (VAP) and pyogenic liver abscess (PLA)	Liver abscess: PLA group: 25/25 (100%); Metastatic spread: not explicitly reported as number of events/total HvKp; Septic shock: not explicitly mentioned	Carbapenem (Imipenem): PLA (0%), VAP (21.24%); Carbapenem (Meropenem): PLA (4%), VAP (28.57%); MDR: PLA (48%), VAP (78.57%); ESBL: PLA (~ 4%), VAP (~ 30%)	PLA group: 1/25 (4%); VAP group: 4/14 (26%)
51	Khairuddin et al.; 2023; Malaysia; Cross-sectional, retrospective (June 2020–June 2021) [65]	Total Kp: 180; HvKp: 17 (9.4%)	Mean: 49.63 years (range 1–85)	HvKp was defined in this study as *K*. *pneumoniae* with a positive string test and harbouring the serotype K1 or K2 gene	NA	Liver abscess: 1/17 (6%); Metastatic spread: NA; Septic shock: NA	Carbapenem: NA; MDR: NA; ESBL: NA	2/17 (11.8%)
52	Jin et al.; 2023; China; Retrospective (May 2018-Aug 2021) [26]	Total Kp: 203; HvKp: 90 (HmKp, string test positive) (44.3%)	Median: 55 years (IQR 48, 63)	Strains exhibiting a mucoid string with a length of ≥ 5 mm were defined as HmKp	NA	Liver abscess: 24/90 (26.7%); Metastatic spread: NA; Septic shock: NA	Carbapenem: 1/90 (1.1%) (HmKp); MDR: NA; ESBL: SHV-type ESBL genes higher in HmKp	NA
53	Yadav et al.; 2023; India; Cross-sectional (Aug 2019–June 2021) [66]	Total Kp: 1004; HvKp: 33 (3.3%)	Predominantly adults 18–60 years (66.7%); 18.1% > 60 years; 15.1% < 18 years	hvKP isolates were identified using the string test	NA	Liver abscess: not specified; Metastatic spread: not specified; Septic shock: not specified	Carbapenem: 10/33 (30.3%); MDR: NA; ESBL: NA	10/33 (30.3%)
54	Li et al.; 2021; China; Descriptive (2017–2019) [67]	Total Kp: 319; HvKp: 26 (8.2%)	Dominated by 6–18 years old (57.7%)	Aerobactin-positive (iucA-positive tested by PCR) *K*. *pneumoniae* was defined as hvKp	NA	Liver abscess: 1/26 (3.8%); Metastatic spread: NA; Septic shock: NA Liver abscess CS-HvKp 1/18 (5.5%)	Carbapenem: 8/26; MDR: NA; ESBL: NA	1/26 (3.8%) CS-HvKp 1/18 (5.5%) CR-HvKp 0/8 (0%)
55	Togawa et al.; 2020; Japan; Retrospective cohort (Apr 2009–May 2019) [68]	Total Kp: 222; HvKp: 10 (defined by liver abscess) (5%)	Median: 72.6 years (range 69.3–75.8) for community-onset BSIs (all HvKp cases)	Hypervirulent *K*. *pneumoniae* infections was defined as infections by *K*. *pneumoniae* associated with liver abscess formation	Bacteremia with liver abscess	Liver abscess: 10/10 (100%); Metastatic spread: NA; Septic shock: NA	Carbapenem: 0/10 (0%); MDR: NA; ESBL: 0/10 (0%)	3/10 (30%)
56	Lin et al.; 2020; Taiwan; Retrospective multi-center (Jan 2013–May 2018) [19]	Total Kp: NA; Total HvKp: 218 KPLA episodes 13 MDR-HvKp	Mean age, 76.00 ± 9.40 years (MDR-HV group)	HvKp strains were defined as those with a hypermucoviscosity phenotype and containing rmpA or rmpA2	Klebsiella pneumoniae liver abscess (KPLA)	Liver abscess: 218/218 (100%); Metastatic spread: NA; Septic shock: 3/13 (23%) (MDR-HV group)	Carbapenem: generally susceptible, MDR: 13 strains; ESBL: 2/13 (15.4%) strains had ESBL genes (blaSHV-5, blaSHV-12); AmpC: 2 strains (blaDHA-1, blaCMY-2); Efflux pumps (AcrAB/OqxAB): 8 strains	In-hospital mortality: 1/13 (7.7%) (MDR-HV group); Infection-related mortality: 1/13 (7.7%) (MDR-HV group)
57	Kim et al.; 2020; Republic of Korea; Prospective (2017–2018) [69]	Total Kp: NA; Total HvKp: 37 patients (11 enrolled for stool analysis)	Median age, 71 years (IQR 50–75 years)	HvKP was confirmed if there was a positive string test	Primary hvKP liver abscess	Liver abscess: 37/37 (100%); Metastatic spread: 2/11 (18.2%); Septic shock: NA	Carbapenem: NA; MDR: NA; ESBL: liver isolates: 0/37 (0%); Stool isolates (non-K1/K2): 8/8 (100%) ESBL	30-day mortality: 0/11 (0%)
58	Tang et al.; 2020; Southwestern China; Retrospective (Feb 2018–June 2019) [28]	Total Kp: 613; Total HvKp: 135 (22%)	Mean age, 60.4 ± 17.1 years (Total HvKp); Non-survivor: 67.4 years; Survivor: 59.5 years	HvKP strains positive for iucA and string test	NA	Liver abscess: NA; Metastatic spread: NA; Septic shock: 1/5 (20%) (CR-HvKp patient)	Carbapenem: 5/135 (3.7%) CR-HvKp (blaKPC-2); MDR: NA ESBL: 13/135 (9.6%) ESBL-producing (blaCTX-M, blaSHV, blaTEM)	In-hospital mortality: 16/135 (11.9%)
59	Li et al.; 2020; Southern China; Retrospective (Jan 2018–Dec 2018) [70]	Total Kp: 495; Total HvKp: 81 (16.4%)	Mean age, 59 ± 17.03 years	HvKP was defined based on a positive wire drawing test and a positive virulence gene (rmpA/rmpA2/iroN genes, and/or positivity of the aerobactin gene)	NA	Liver abscess: 13/81 (16%) (from drainage); Metastatic spread: NA Septic shock: NA;	Carbapenem: 0/81 (0%); MDR: NA; ESBL: 6/81 (7.4%)	7/66 (10.6%) 15 Abandoned treatment
60	Zhou et al. (2021; China; Retrospective; 2019–2020) [71]	Total Kp: 1081; Total CR-hvKP: 16 (1.5%)	Mean age: 83.1 ± 10.5 years (all patients older than 62 years)	Hypervirulent *Klebsiella* *pneumoniae* is defined as having a hypermucoviscous phenotype and carrying *K*. *pneumoniae* virulence plasmid-associated loci (rmpA2, iutA, iucA)	NA	Liver abscess: NA; Metastatic spread: NA; Septic shock: NA	Carbapenem: 16/16 (100%) resistant to imipenem & meropenem; all carry *blaKPC-2* MDR: NA ESBL: all 16 carried *blaSHV*; 5 carried *blaCTX-M-1* group; 6 carried *blaCTX-M-9* group	9/16 (56.3%)
61	Hyun et al. (2019; Korea; Retrospective; 2013–2015) [72]	Total Kp: 414; Total HvKP: 155 (37.4%)	Mean age: 67.8 ± 13 years (for all study participant)	Hypervirulence was determined by the presence of a hypermucoviscous phenotype	Health Care-Associated infections	Liver abscess: NA; Metastatic spread: NA; Septic shock: not quantified	Carbapenem: 0/155 (0%) resistance MDR: higher in health care-associated hvKP. Cephalosporins ~ 20% resistant ESBL: 15/155 (9.6%) overall; Health care-associated: 12/60 (20%); Community-acquired: 3/95 (3.2%)	Health care-associated HvKP: 13/60 (21.7%) Community-origin HvKP: 8/95 (8.4%) Total: 21/155 (13.5%)
62	Chen et al. (2018; China; NA) [73]	Total Kp: NA; Total HMKP: 42	NA	Hypervirulence was determined by the presence of a hypermucoviscous phenotype	Health Care-Associated infections	Liver abscess: NA; Metastatic spread: NA; Septic shock: NA	Carbapenem: 10/42 (23.8%) CR-HMKP, produced *KPC-2* MDR: CR-HMKP resistant to carbapenems, high MICs for amikacin, gentamicin, ciprofloxacin, levofloxacin, trimethoprim/sulfamethoxazole ESBL: not detected among CS-HMKP	CR-HMKP: 6/10 (60.0%) CS-HMKP: 2/32 (6.3%)
63	Zhan et al. (2017; China; Retrospective; 2013–2015) [74]	Total Kp: 1838; Total HMKP (CRKP): 21 (1.1%)	22–82 years old (range)	Hypervirulence was determined by the presence of a hypermucoviscous phenotype	NA	Liver abscess: NA; Metastatic spread: NA; Septic shock: 1/21 (4.8%)	Carbapenem: 21/21 (100%) resistant to imipenem. All positive for *blaKPC-2* MDR: 100% resistant to ampicillin, ampicillin/sulbactam, aztreonam, cefazolin, piperacillin/tazobactam, ceftriaxone. High resistance rates for other agents ESBL: 15/21 (71.4%) harbored *blaCTX-M-65*	1/21 (4.8%) directly died 11/21 (52.4%) (refused further treatment due to uncontrolled infections
64	Xiao et al.; 2017; China; Retrospective (Jan 2013–Jun 2014) [75]	Total Kp: 533; Total HvKp: 24 (infection isolates) (4.5%)	Not reported (only > 60 years: Group 1: 80.0%, Group 2: 44.4%)	HvKp was defined by phenotype (string test > 5 mm)	NA	**Overall:** Liver abscess: NA (Group 1), 6/9 (66.7%) (Group 2); Metastatic spread: 0/15 (0%) (Group 1), 1/9 (11.1%) (Group 2); Septic shock: NA	**Overall:** Carbapenem resistance: 2/24 (8.3%) (2/15 (13.3%) in Group 1, 0/9 (0%) in Group 2), specifically Imipenem and Meropenem; MDR: NA; ESBL: NA	Total: 4/24 (16.7%) (Group 1: 3/15 (20.0%); Group 2: 1/9 (11.1%))
65	Ye et al.; 2016; China; Clinical, molecular, and genomic sequencing analyses (Jan 2014–Jan 2016) [18]	Total Kp: 40 (LA-Kp); Total HvKp: 40 (LA-Kp) (100%) String test was positive for 28 isolates	Average age: 59.3 (33–81) years	Genotype	Pyogenic liver abscess (KLA)	Liver abscess: 40/40 (100%) (HM-isolates 28); Metastatic spread: NA; Septic shock: NA	Carbapenem resistance: 0/40 (0%); MDR: NA; ESBL: NA	0/40 (0%)
66	Zhou et al.; 2024; China; Retrospective observational (Jan 2014–Jan 2021) [15]	Total Kp: 39 patients; Total HvKp: 16 isolates (3 avirulent CRKP) (41%)	Mean ± SD 53.3 ± 14.9 years (range 16–85)	HvKP was defined mentioned molecular markers (rmpA, rmpA2, iucA, iroB, peg-344) and mouse lethality assay	Endogenous *Klebsiella pneumoniae* endophthalmitis (EKPE)	Liver abscess: 28/39 (71.8%); Metastatic spread: NA; Septic shock: 8/39 (20.5%)	Carbapenem resistance: 3/16 (18.8%); MDR: NA; ESBL: NA	4/39 (10.3%)
67	Huang et al.; 2022; China; Retrospective (2014–2020) [76]	Total Kp: 36; Total HvKP: 24/36 (66.7%); Total Hv-CRKP: 13/36 (36.1%)	Average age: 54 years (IQR 44, 62)	HvKP was defined by genotype (peg-344 and iucA carrying strains)	Meningitis (*K*. *pneumoniae* meningitis)	Overall Liver abscess: 2/36 (5.6%), CR-HvKp-0/13 (0%); Metastatic spread: NA; Septic shock: NA	Carbapenem resistance: 13/24 (54.16%); MDR: NA; ESBL: NA	HvKp-18/24 (75%) CR-HvKp 12/13 (92.3%) Non-Hv-CRKP (CR-cKp) 13/23 (56.5%)
68	Kamau et al.; 2022; USA; Passive surveillance (Sept 2020–Mar 2022) [77]	Total Kp: 567 (invasive); Total HvKp: 15 (2.6%)	Ranged from 30 to 82 years (median age 55)	HvKp was defined by genotype (≥ 3 of iucA, iroB, peg-344, rmpA, rmpA2 detected with > 99% full-length gene coverage)	Invasive *Klebsiella pneumoniae* infections (including hepatic abscess, lung, neck, pelvis, eye infections)	Liver abscess: 6/15 (40%); Metastatic spread: NA (various sites of infection reported, not as metastatic events from a primary source); Septic shock: NA	Carbapenem resistance: 0/15 (0%); MDR: 0/15 (0%); ESBL: 0/15 (0%)	2/15 (13.3%)
69	Zhang et al.; 2019; China; Retrospective (Jan 2016–Dec 2017) [78]	Total Kp: NA; Total HvKp: 163 (KP-PLA)	Median 63.0 years (52.3–70.0)	HvKp was defined by hypermucoviscosity (string test > 5 mm)	Pyogenic liver abscess	Liver abscess: NA; Metastatic spread: NA; Septic shock: 15/163 (9.2%)	Carbapenem resistance: 6/163 (3.7%); MDR: 12/163 (7.4%); ESBL: NA	1/163 (0.6%)
70	Zhao et al.; 2019; China; Outbreak investigation (March 2017–Jan 2018) [79]	Total Kp: 29 (isolates from 8 patients) (27.6%); Total CR-HvKp: 8/8 (100%)	Patient age range 13–69 years	HvKp was defined by genotype (positive for 19 virulence-associated genes including iutA, rmpA, ybtA, entB, fimH, mrkD)	Nosocomial infections in ICU patients	Liver abscess: NA; Metastatic spread: NA; Septic shock: 8/8 (100%) (all patients died of septic shock and multiple organ failure)	Over all isolates Carbapenem resistance: 28/29 (96.6%); MDR: 28/29 (96.6%); ESBL: *blaSHV-11* (28 isolates), *blaCTX-M-2 group* (27 isolates), *blaCTX-M-9 group* (23 isolates), *blaTEM-1* (10 isolates), *blaCMY-2* (7 isolates), *blaDHA* (4 isolates)	8/8 (100%)
71	Lin et al. (2018; Taiwan; Retrospective multi-centre study between April 2013 and March 2016) [80]	Total Kp: 182 K1 strains; Total HvKp: 182 K1 strains (100%)	NA	Capsular type K1 strain; all showed hypermucoviscosity phenotypes and carried rmpA	NA	Liver abscess: 54/182 (29.7%); Metastatic spread: 16/182 (8.8%); Septic shock: 59/182 (32.4%)	NA	26/182 (14.3%)
72	Kim et al. (2019; South Korea; Prospective multicentre observational study from May 2016 to April 2017) [81]	Total Kp: 579; Total HvKp: 191 (33%) (hypermucoviscous phenotype)	NA	Hypermucoviscous phenotype	Bacteremia	Liver abscess: NA; Septic shock: not specified; Metastatic spread: not specified	NA	24/191 (12.6%) With HM-phenotype
73	Candra et al. (2023; Indonesia; Retrospective cross-sectional study from December 1, 2020, to May 31, 2021) [82]	Total Kp: 51; Total HvKp: 5 (9.8%)	Average age of 49 years. All 5 HvKp patients were > 18 years old (100%)	Positive PCR of *rmpA, iucA* genes, AND/OR a positive string test (> 5 mm); defined by ≥ 2 of 3 markers (string test, *rmpA*, *iucA*)	Bacteremia	Liver abscess: not specified; Metastatic spread: not specified; Septic shock: 4/5 (80%)	Carbapenem Resistance: 0% (0/5); MDR: 0% (0/5); ESBL: 0% (0/5)	5/5 (100%)
74	Yang et al. (2022; China; Retrospective cohort study from January 2017 to March 2021) [83]	Total Kp: 139; Total HvKp: 75 (54%); Total CR-HvKp: 31 (41.3%)	NA	HvKp is defined based on the combination of *peg-344,iroB, iucA, rmpA, or rmpA2* positivity	NA	Liver abscess: NA; Septic shock: not specified; Metastatic spread: 15/75 (20%)	Carbapenem Resistance: 41.3% (31/75); MDR: 32/75 (42.6%) ESBL: not specified	HvKp: 14/73 (19.1%) (2 patients data missing); CR-HvKp: 10/31 (32.3%); CS-HvKp: 4/42 (9.5%)
75	Zhang et al. (2022; China; Retrospective study from 2017 to 2020) [84]	Total Kp: 185 Total CR-Kp: 146 (79%) Total CR-HvKp:15 (10.3%)	NA	Hypermucoviscous phenotype	Bacteremia	Liver abscess: NA; Septic shock: not specified; Metastatic spread: not specified	Carbapenem Resistance: 100% (CR-HvKp focused); MDR: not specified ESBL: not specified	CR-HvKp: 10/15 (66.7%)
76	Wei et al. (2022; China; Retrospective study from January 2020 to December 2020) [85]	Total: 80 CRKP Total; 51 CR-HvKp (63.8%)	Median 85 (75–88)	HvKp is defined based on the presence of anyone of the virulence genes *peg-344,iroN, iucA, rmpA, or rmpA2* positivity	NA	Liver abscess: 4/51 (7.8%); Septic shock: not specified; Metastatic spread: not specified	Carbapenem Resistance: 100% (CR-HvKp focused); MDR: not specified ESBL: not specified	21/51 (41.2%)
77	Shankar et al. (2018; India; Retrospective study from 2014 to 2015) [86]	Total CR-Kp: 86 Total CR-HvKp: 27 (31.4%)	NA	Hypermucoviscous phenotype	Bacteremia	Liver abscess: not specified; Septic shock: not specified; Metastatic spread: not specified	Carbapenem Resistance: 100% (CR-HvKp focused); MDR: not specified ESBL: not specified	20/27 (74.1%)
78	Ouyang et al. (2022; China; Retrospective study January 2018-December 2018) [87]	Total Kp: 1743 Total CR-Kp: 125 (7.2%) Total CR-HvKp: 41 (33%)	NA	HvKp is defined based on the presence of the virulence genes *iroN, iucA, rmpA, or rmpA2* positivity	Nosocomial infections	Liver abscess: not specified; Septic shock: not specified; Metastatic spread: not specified	Carbapenem Resistance: 100% (CR-HvKp focused); MDR: not specified ESBL: not specified	7/41 (7.1%)
79	Pan et al. (2019; China: Retrospective study 2014) [88]	Total CR-Kp: 66 Total CR-HvKp: 15 (22.7%)	NA	Hypermucoviscous phenotype	Healthcare associated infections	Liver abscess: not specified; Septic shock: not specified; Metastatic spread: not specified	Carbapenem Resistance: 100% (CR-HvKp focused); MDR: not specified ESBL: not specified	9/15 (60%)

*AMR* antimicrobial resistance, *BLA* beta-lactamase, *blaCMY* CMY-type AmpC beta-lactamase, *blaCTX-M* Cefotaximase Munich, extended-spectrum beta-lactamase, *blaDHA* DHA-type AmpC beta-lactamase, *blaKPC/blaKPC-2* *Klebsiella pneumoniae* carbapenemase, *blaNDM/blaNDM-1* New Delhi Metallo-beta-lactamase, *blaSHV* sulfhydryl variable beta-lactamase, *blaTEM* Temoneira beta-lactamase, *BSI* bloodstream infection, *CA* community-acquired, *CARB* carbapenem, *CKp/cKp* classical *Klebsiella pneumoniae*, *CLA* community-acquired liver abscess, *CR-HMKP* carbapenem-resistant hypermucoid *Klebsiella pneumoniae*, *CR-HvKp/CR-HvKP* carbapenem-resistant Hypervirulent *Klebsiella pneumoniae*, *CRKP/CR-Kp* carbapenem-resistant *Klebsiella pneumoniae*, *CS-HMKP* carbapenem-susceptible hypermucoid *Klebsiella pneumoniae*, *CS-HvKp* carbapenem-susceptible Hypervirulent *Klebsiella pneumoniae*, *CTX-M* Cefotaximase Munich, extended-spectrum β-lactamase gene family, *EKPE* endogenous *Klebsiella pneumoniae* endophthalmitis, *entB* Enterobactin biosynthesis gene B, *ESBL* extended-spectrum beta-lactamase, *fimH* Type 1 fimbrial adhesin, *HA* hospital-acquired, *HM/Hm/HMKP* hypermucoviscosity or hypermucoviscous *Klebsiella pneumoniae*, *HMKp/HmKp* hypermucoviscous *Klebsiella pneumoniae*, *HvKp/hvKP/hvKp/HvKP/hvKp-p* Hypervirulent *Klebsiella pneumoniae*, *ICU* intensive care unit, *iucA* aerobactin synthetase gene, *iutA* ferric aerobactin receptor gene, *IQR* interquartile range, *iroB* salmochelin glucosyltransferase gene, *KLA/KPLA* *Klebsiella pneumoniae* liver abscess, *KP/Kp* *Klebsiella pneumoniae*, *LA-Kp* liver abscess-associated *Klebsiella pneumoniae*, *magA* mucoviscosity-associated gene A, *MDR* multidrug resistant, *MIC* minimum inhibitory concentration, *mrkD* Type 3 fimbrial adhesin, *NA* not available or not applicable, *NDM* New Delhi Metallo-beta-lactamase, *peg-344* putative metabolite transporter gene on virulence plasmid, *PLA* pyogenic liver abscess, *prmpA/prmpA2/p-rmpA* plasmid regulator of mucoid phenotype A and A2, *rmpA* regulator of mucoid phenotype A, *rmpA2* regulator of mucoid phenotype A2, *SD* standard deviation, *SHV* sulfhydryl variable beta-lactamase gene, *ST23* sequence Type 23, multilocus sequence type, *TEM* Temoneira beta-lactamase, *VAP* ventilator-associated pneumonia, *ybtA* yersiniabactin biosynthesis gene A

### Liver abscess

**Fig. 3:**
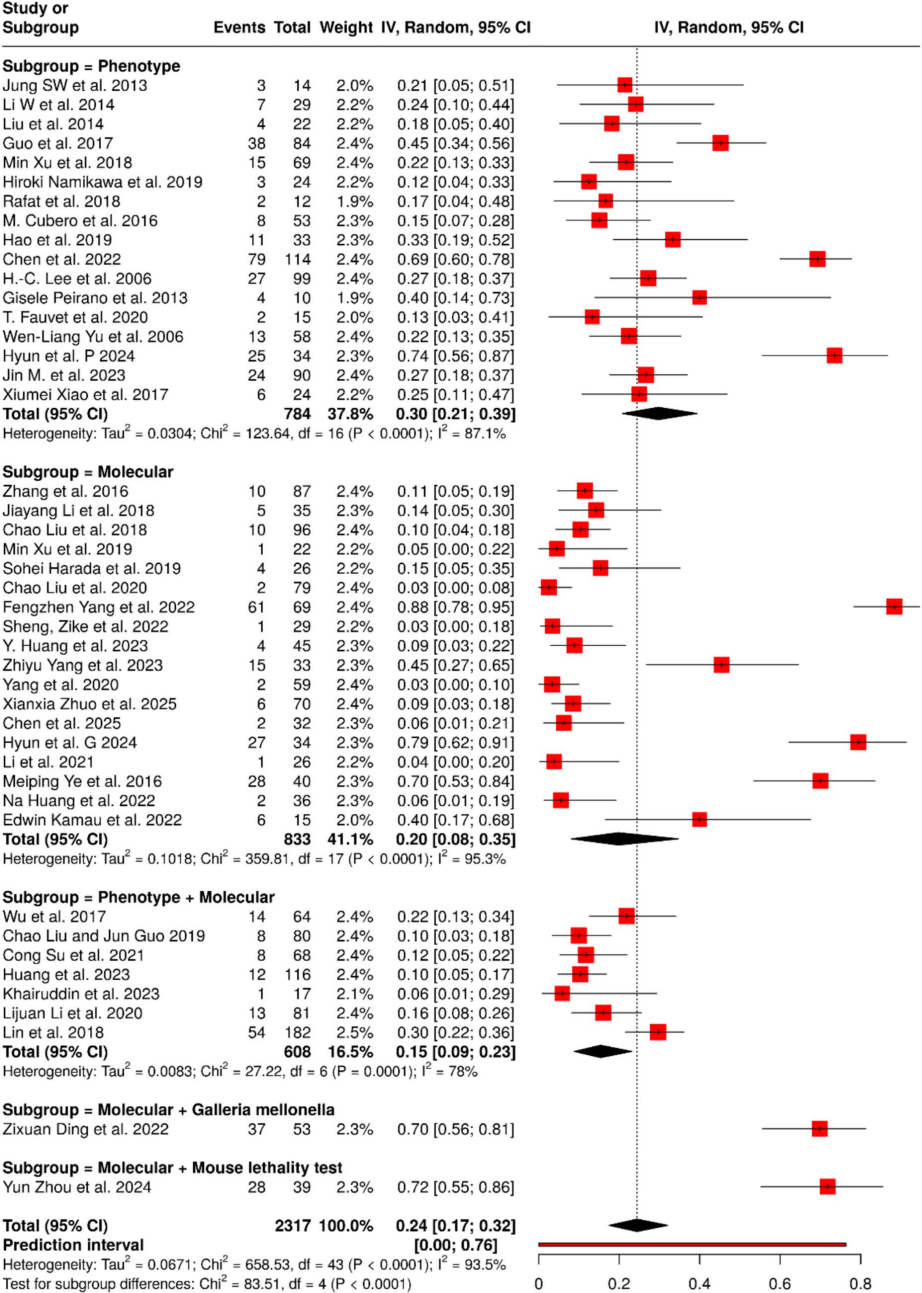
Pooled frequency of liver abscess among HvKp infected patients with subgroup analysis

In 44 cohort studies comprising 2317 patients with HvKp, the pooled proportion of liver abscess was 24% (95% CI 17–32) with substantial heterogeneity (I^2^ = 93.5%, p < 0.0001) (Fig. 3). Subgroup analysis demonstrated that the method used to define HvKp markedly influenced proportion estimates: phenotypic criteria (18 studies; n = 784) yielded a proportion of 30% (95% CI 21–39; I^2^ = 87.1%), molecular criteria (20 studies; n = 833) showed 20% (95% CI 8–35; I^2^ = 95.3%), and combined phenotypic plus molecular criteria (11 studies; n = 608) yielded 15% (95% CI 9–23; I^2^ = 87.7%). Single studies using molecular plus *Galleria mellonella* infection model (n = 53) and molecular plus mouse lethality test (n = 39) reported much higher proportions of 70% (95% CI 56–81) and 72% (95% CI 55–86), respectively. Between-subgroup differences were statistically significant (Chi^2^ = 83.51, df = 4, p < 0.0001), indicating that definitional criteria substantially influence the reported proportion of liver abscess in HvKp.

### Metastatic spread

**Fig. 4:**
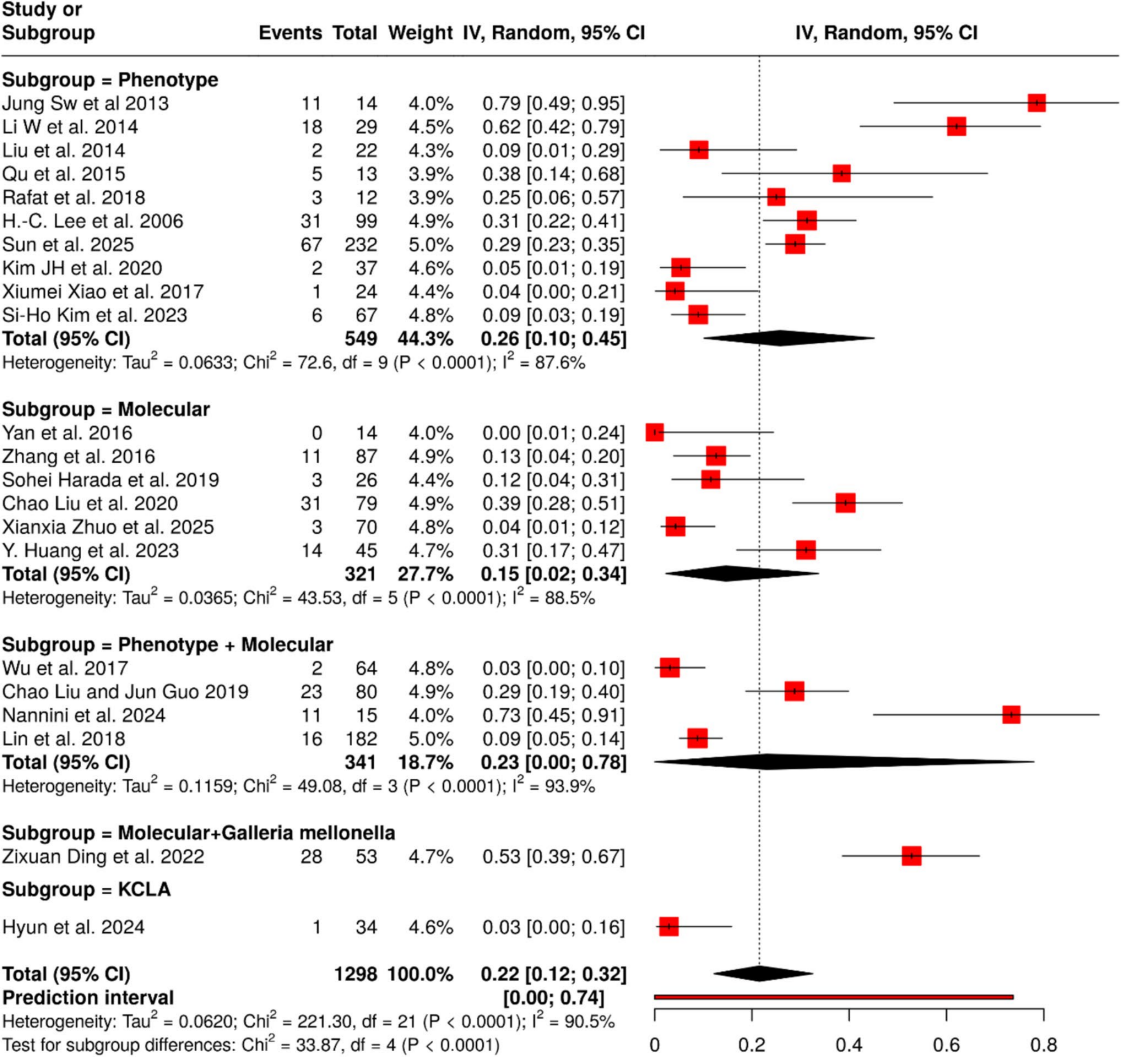
Pooled frequency of metastatic spread of infections among HvKp infected patients with subgroup analysis

In 22 studies including 1298 patients with HvKp, the pooled proportion of metastatic spread was 22% (95% CI 12–32) with substantial heterogeneity (I^2^ = 90.5%, p < 0.0001) (Fig. 4). Subgroup analysis by the method of HvKp definition showed variability in estimates: phenotypic criteria (10 studies; n = 549) yielded a proportion of 26% (95% CI 10–45; I^2^ = 87.6%), molecular criteria (6 studies; n = 321) showed 15% (95% CI 2–34; I^2^ = 88.5%), and combined phenotypic plus molecular criteria (5 studies; n = 341) yielded 23% (95% CI 0–78; I^2^ = 93.9%). Single studies using molecular plus Galleria mellonella infection model (n = 53) and *K*. *pneumoniae* liver abscess (KCLA) definition (n = 34) reported proportions of 53% (95% CI 39–67) and 3% (95% CI 0–16), respectively. Between-subgroup differences were statistically significant (Chi^2^ = 33.87, df = 4, p < 0.0001), indicating that definitional criteria substantially influence reported proportions of metastatic spread in HvKp.

### Septic shock

**Fig. 5:**
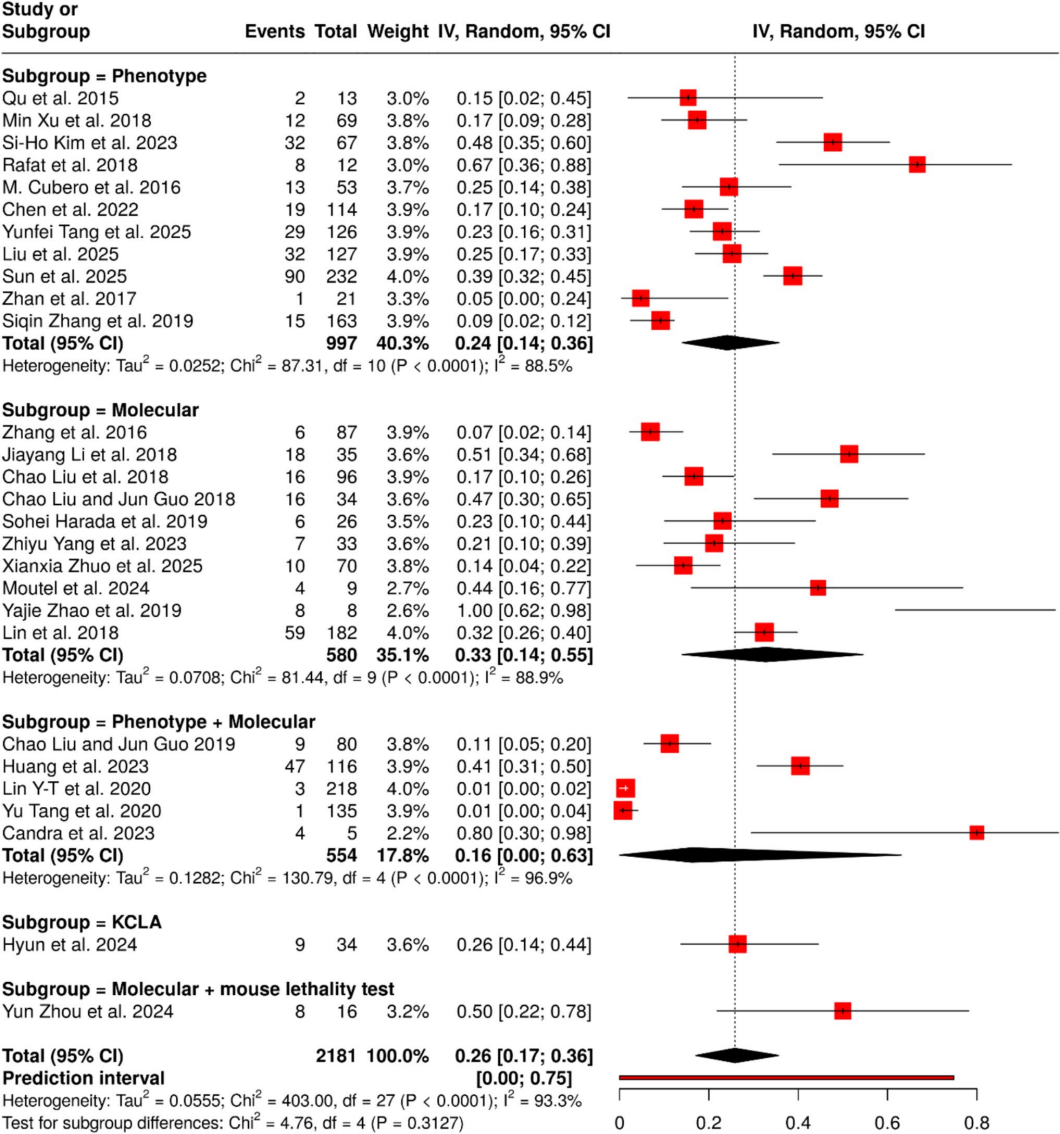
Pooled frequency of septic shock among HvKp infected patients with subgroup analysis

In 28 studies including 2181 patients with HvKp, the pooled proportion of septic shock was 26% (95% CI 17–36) with substantial heterogeneity (I^2^ = 93.3%, p < 0.0001) (Fig. 5). Subgroup analysis by HvKp definition showed variability in estimates: phenotypic criteria (13 studies; n = 997) yielded a proportion of 24% (95% CI 14–36; I^2^ = 88.5%), molecular criteria (11 studies; n = 580) showed 31% (95% CI 14–55; I^2^ = 88.9%), and combined phenotypic plus molecular criteria (4 studies; n = 554) yielded 15% (95% CI 0–61; I^2^ = 96.9%). Single studies using *K*. *pneumoniae* liver abscess (KCLA) definition (n = 34) and molecular plus mouse lethality test (n = 16) reported proportions of 26% (95% CI 14–44) and 50% (95% CI 22–78), respectively. Between-subgroup differences were not statistically significant (Chi^2^ = 4.76, df = 4, p = 0.3127).

### Mortality

**Fig. 6:**
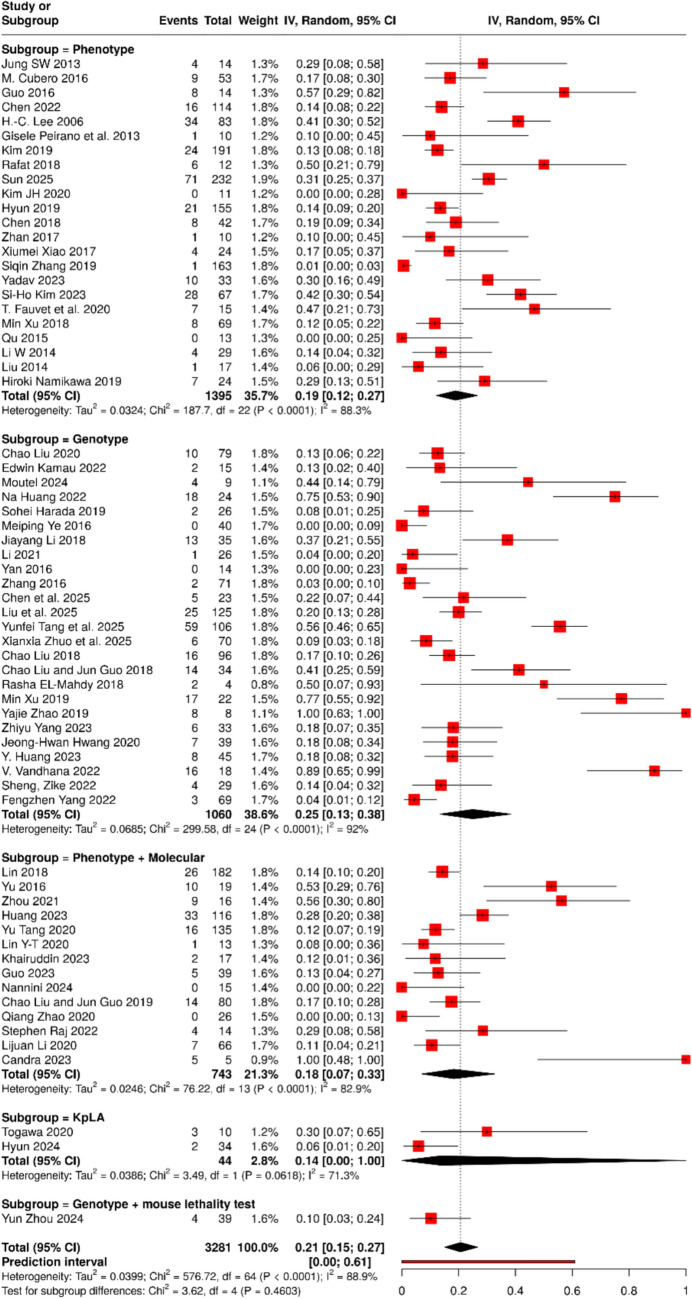
Pooled frequency of in-hospital mortality among HvKp infected patients with subgroup analysis

In 65 studies including 3281 patients with HvKp, the pooled proportion of mortality was 21% (95% CI 15–27) with substantial heterogeneity (I^2^ = 88.9%, p < 0.0001) (Fig. 6). Subgroup analysis by HvKp definition showed variability in estimates: phenotypic criteria (27 studies; n = 1395) yielded a proportion of 19% (95% CI 12–27; I^2^ = 88.3%), genotypic criteria (28 studies; n = 1060) showed 25% (95% CI 13–38; I^2^ = 92%), and combined phenotypic plus molecular criteria (13 studies; n = 743) yielded 18% (95% CI 7–33; I^2^ = 82.5%). Single studies using *K*. *pneumoniae* liver abscess (KpLA) definition (n = 44) and molecular plus mouse lethality test (n = 39) reported proportions of 14% (95% CI 0–100) and 10% (95% CI 3–24), respectively. Between-subgroup differences were not statistically significant (Chi^2^ = 3.62, df = 4, p = 0.4603).

### CR-HvKp mortality proportion

**Fig. 7:**
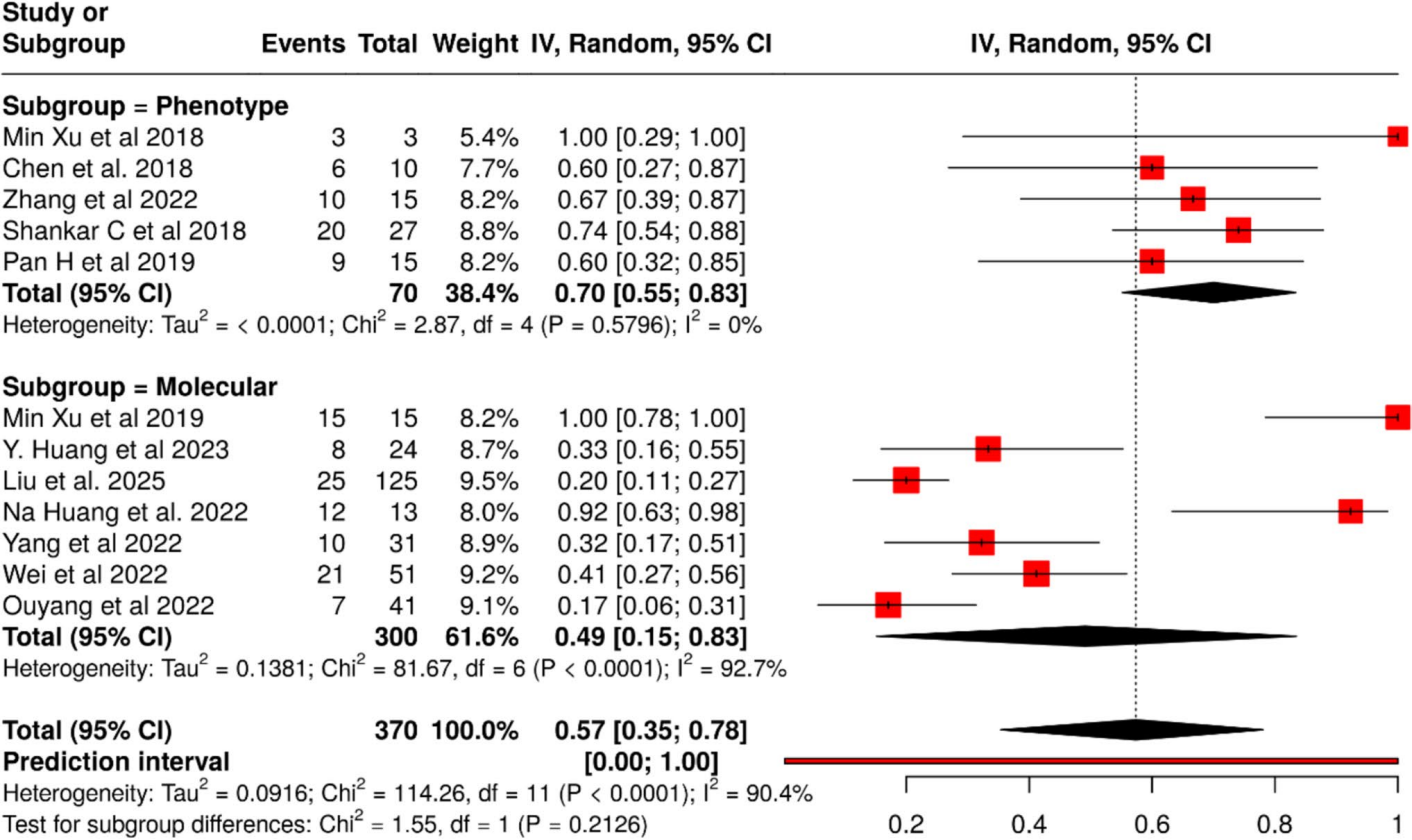
Pooled frequency of in hospital mortality among CR-HvKp infected patients with subgroup analysis

In 11 cohort studies comprising 370 patients with CR-HvKp, the pooled mortality proportion was 57% (95% CI 35–78; I^2^ = 90.4%, p < 0.0001) (Fig. 7), with subgroup analysis showing higher mortality when defined by phenotypic criteria (70%; 95% CI 55–83; I^2^ = 0%) compared to molecular criteria (48%; 95% CI 15–83; I^2^ = 92.7%), though the between-subgroup difference was not statistically significant (p = 0.2126).

### Comparison of mortality risk with CR-HvKp vs CS-HvKp

**Fig. 8:**
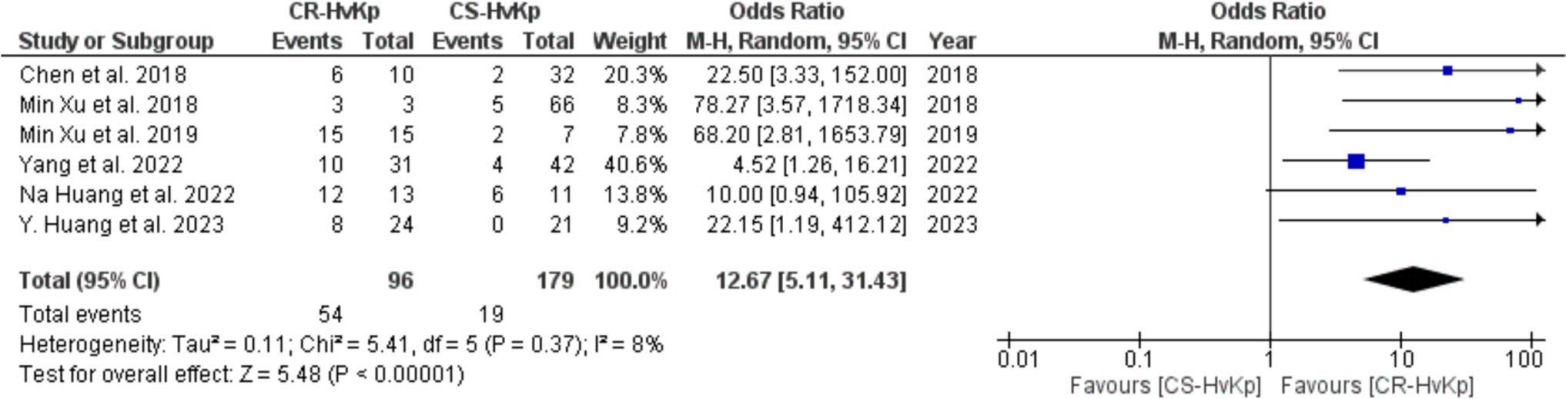
Odds of mortality among CR-HvKp vs CS-HvKp infected patients

Meta-analysis of six studies comparing CR-HvKp with CS-HvKp demonstrated a strong and statistically significant association between carbapenem resistance and mortality (pooled OR = 12.67, 95% CI 5.11–31.43; p < 0.00001) (Fig. 8). These studies included a total of 96 patients in the CR-HvKp group and 179 patients in the CS-HvKp group. The direction and magnitude of effect were consistent across all studies, with only minimal statistical heterogeneity (I^2^ = 8%). The high precision of the pooled estimate and the consistency across studies strengthen the robustness of this finding, underscoring the substantial mortality risk conferred by carbapenem resistance in HvKp infections.

### Rate of microbiological failure among HvKp infected patients

**Fig. 9:**
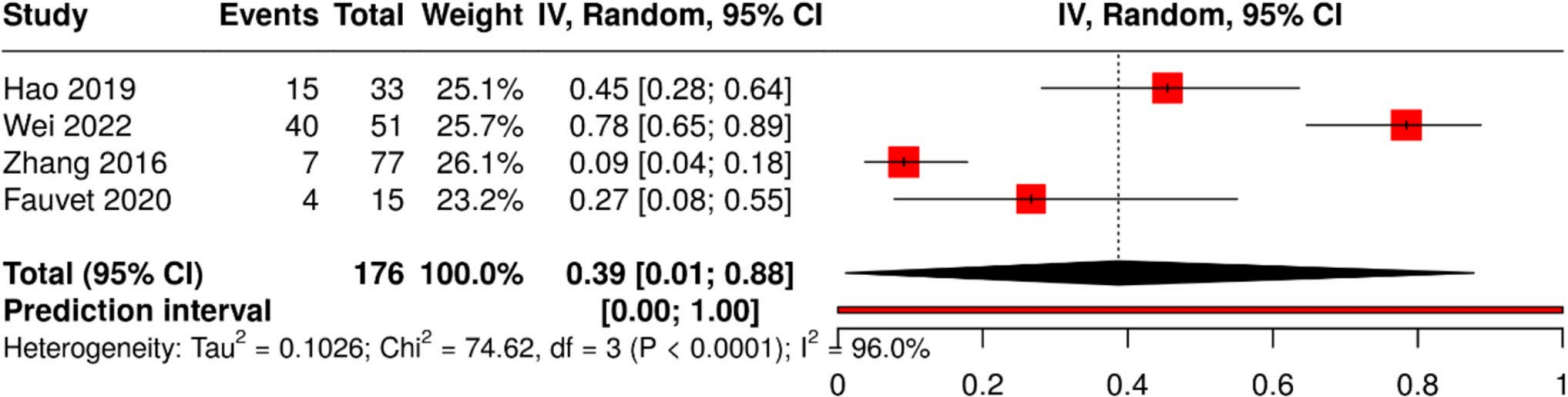
Pooled frequency of microbiological failure among HvKp infected patients

Across four studies comprising 176 patients with HvKp, the pooled rate of microbiological failure was 39% (95% CI 1–88; I^2^ = 96%, p < 0.0001) (Fig. 9). Among these, two studies defined HvKp using molecular criteria, one employed a combination of phenotypic and genotypic markers, and one relied solely on phenotypic definition.

### Critical appraisal of literature

Most studies (> 90%) clearly defined their inclusion criteria and described their subject populations and settings, ensuring strong contextual relevance (Supplementary Table 1). Similarly, more than 90% measured exposures and conditions in a valid and reliable manner, reflecting high methodological consistency. Nearly all studies (> 90%) employed appropriate statistical analyses aligned with their objectives. These findings indicate a generally robust methodological standard across the included literature, with only minimal gaps in statistical reporting.

### Publication bias and sensitivity analysis

**Fig. 10:**
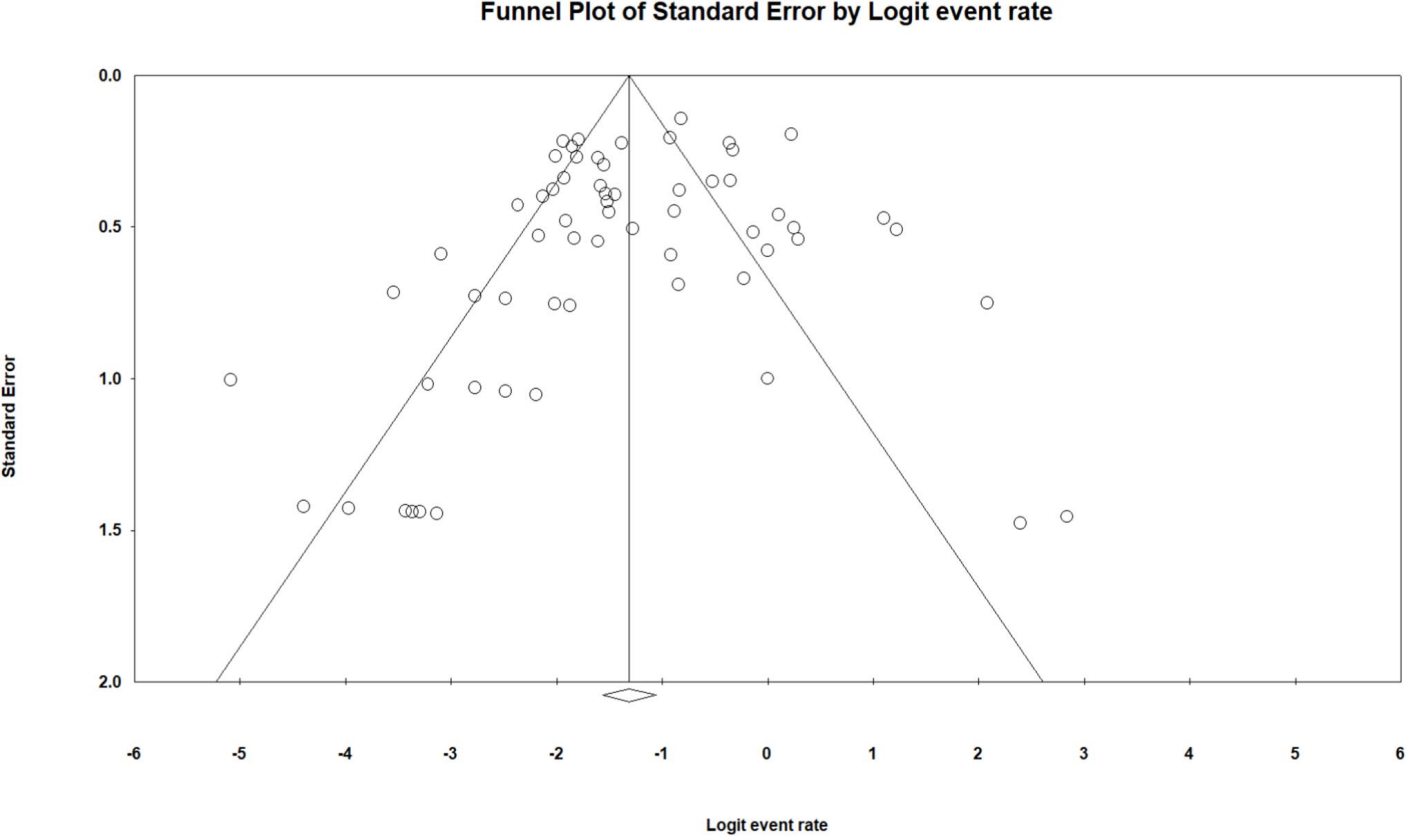
Funnel plot assessing publication bias

Publication bias was evaluated using a funnel plot of standard error by logit event rate (Fig. 10). The plot appeared largely symmetric, suggesting minimal evidence of bias. Begg and Mazumdar’s rank correlation test showed no significant association between study size and effect size (Kendall’s tau b = − 0.040, p = 0.63), while Egger’s regression test was also non-significant (intercept = − 0.858, 95% CI − 2.01 to 0.29, p = 0.14), indicating no evidence of small-study effects.

Sensitivity analysis demonstrated that sequential exclusion of individual studies did not materially alter the pooled estimates, and results remained consistent under both fixed- and random-effects models. These findings confirm the robustness of the results and suggest that the observed effect is not driven by publication bias or by any single study.

## Discussion

This systematic review and meta-analysis highlights the dual threat posed by HvKp, its intrinsic invasive potential and the escalating problem of antimicrobial resistance. Two major insights emerge. First, the absence of a standardized diagnostic definition remains a significant driver of heterogeneity across studies, complicating efforts to quantify HvKp’s true clinical burden. Second, and most concerning, is the convergence of hypervirulence with carbapenem resistance, which markedly increases the risk of death in infected patients.

### Clinical implications

Previous analyses have reported that although HvKp infections are more likely to cause invasive complications, mortality rates have not consistently exceeded those seen with cKp infections [4]. Our findings refine this understanding by demonstrating that the acquisition of carbapenem resistance substantially alters this relationship. Once resistance emerges, mortality rises sharply- the pooled mortality for CR-HvKp reached 57%, nearly three times higher than the 21% observed in the overall HvKp population. A direct comparison revealed more than a 12-fold higher odds of death among patients with CR-HvKp compared to those with CS-HvKp, with minimal heterogeneity across studies. These results underscore a lethal synergy in which virulence factors promote rapid invasion and dissemination while resistance compromises treatment efficacy, leaving few effective and often toxic therapeutic options. The convergence of hypervirulent traits with multidrug resistance represents a high-risk trajectory, a potential ‘superbug in the making,” highlighting the urgent need for early recognition, genomic surveillance, and strict antimicrobial-stewardship measures to prevent the establishment and dissemination of such convergent clones.

Liver abscess and metastatic spread remain the hallmark clinical manifestations of HvKp infection. Our meta-analysis confirmed that these complications occur most frequently in strains exhibiting the HMV- phenotype. This phenotype, regulated by *rmpA* and *rmpA2*, promotes excessive capsular polysaccharide production and confers enhanced resistance to phagocytosis and complement-mediated killing [2, 11–14, 23–25]. Other virulence determinants including magA (K1 capsular gene cluster), kfu (iron acquisition), allS (allantoin metabolism), pagO, and siderophore systems like aerobactin (*iucA*) and salmochelin (*iroB*) further promote hepatic invasion, iron scavenging, and systemic dissemination [26–30]. Diabetes mellitus is a consistently identified host risk factor, particularly for pyogenic liver abscesses, likely due to impaired neutrophil function, compromised intestinal barrier integrity, and hyperglycemia-induced CPS overproduction [12, 31, 32]. Importantly, HvKp infections can occur in otherwise healthy individuals, reflecting the pathogen’s intrinsic virulence.

A primary contribution of this meta-analysis is the quantification of how diagnostic ambiguity distorts our understanding of HvKp-associated risks. The significant between-subgroup heterogeneity for liver abscess (p < 0.0001) and metastatic spread (p < 0.0001) confirms that the choice of identification method is a primary driver of outcome variability. Studies employing the string test (HMV- phenotypic) reported a higher proportion of liver abscess (30%) compared to those using molecular markers alone (20%). This may reflect a historical bias, where the string test was preferentially used in cohorts with classic invasive syndromes like pyogenic liver abscess. Conversely, definitions requiring both phenotypic and molecular confirmation yielded the lowest proportion of liver abscess (15%), suggesting such stringent criteria may lack sensitivity and exclude genuinely virulent isolates that do not express both sets of markers. The highest proportions of liver abscess (72%) and metastatic spread (53%) were observed in studies that incorporated animal models, the experimental gold standard for virulence assessment. This finding supports the superior predictive accuracy of in vivo assays for true virulence. However, their cost, complexity, and ethical constraints render them impractical for routine clinical diagnostics or large-scale surveillance. This highlights the translational gap between research-level virulence assays and routine clinical diagnostics. The lack of a consensus definition is therefore not merely a semantic issue but a primary confounder that permeates the literature, contributing directly to the high statistical heterogeneity (I^2^ > 88%) observed across all our pooled estimates and limiting the comparability of epidemiological data worldwide.

Despite the definitional variability, this meta-analysis confirms the substantial clinical burden of HvKp. The pooled proportion of 24% for liver abscess reinforces the classic association of HvKp with this severe syndrome, often in the absence of underlying hepatobiliary disease. The 22% pooled proportion for metastatic spread quantifies one of the most feared hallmarks of HvKp infection, its ability to disseminate from a primary site to cause devastating complications such as endophthalmitis, meningitis, and distant abscesses, even in healthy hosts. The high rate of septic shock (26%) further reflects the aggressive and invasive nature of these infections.

Interestingly, while the method of identification of HvKp significantly influenced the reported rates of initial invasive events like liver abscess, it did not significantly alter the pooled estimates for septic shock (p = 0.3127) or mortality (p = 0.4603). This suggests a potential two-stage clinical progression. The initial invasion and establishment of infection (e.g., liver abscess) may be strongly dependent on specific virulence factors captured by different diagnostic criteria. However, once a severe, systemic infection is established, the subsequent progression to septic shock and death may be more heavily influenced by a complex interplay of host factors, such as age, comorbidities, and immune response, and the timeliness and appropriateness of clinical management. This would explain why the overall mortality rate of 21%, while high, did not vary significantly based on the laboratory definition of the pathogen. This finding implies that the critical window for intervention is early in the disease course, and diagnostic strategies should prioritize the identification of strains with high invasive potential.

The markedly higher mortality associated with CR-HvKp underscores the dual threat that arises when hypervirulence converges with antimicrobial resistance [3, 7]. Evidence regarding newer β-lactam/β-lactamase inhibitor (BLBLI) combinations further reinforces this concern. In the study by Wei et al., HvKp isolates exhibited low resistance rates to ceftazidime–avibactam (2.0%) and imipenem–avibactam (3.9%). In contrast, Huang et al. reported complete (100%, 8/8 isolates) resistance to ceftazidime–avibactam among strains co-producing NDM-1 and KPC-2,demonstrating that the coexistence of multiple carbapenemases can undermine even advanced BLBLI therapies [7, 85] (Supplementary Table 2).

Clinical outcomes parallel these microbiological findings, Tiseo et al. observed that outcomes with meropenem–vaborbactam were favourable only when organ dysfunction was limited and early source control was achieved. Mortality remained high in patients with severe disease or respiratory involvement despite in-vitro susceptibility [89]. These results support our observation that poor outcomes in CR-HvKp infections stem from an interplay between pathogen virulence, host severity, and the timeliness of management. Similarly, Falcone et al. highlighted the limited efficacy of current BLBLI regimens against metallo-β-lactamase-producing *Enterobacterales*, emphasizing the therapeutic ceiling encountered when virulence and resistance coincide [90].

Collectively, these findings underscore the urgent need for an integrated approach combining rapid molecular diagnostics, prompt source control, and antimicrobial stewardship-guided use of emerging agents to combat the escalating threat of hypervirulent, drug-resistant *K. pneumoniae* lineages.

In addition to high mortality, microbiological failure emerged as another clinically important endpoint. This high rate of persistent positivity despite apparently appropriate antimicrobial therapy suggests that HvKp is particularly difficult to eradicate, possibly due to its biofilm-forming ability, capsular protection, and propensity for device or tissue seeding. These findings emphasize the importance of early source control, removal of infected catheters or drains, and vigilant follow-up cultures in achieving microbiological clearance [14, 52, 57, 85].

Approximately 65% of the studies included in this review originated from China, corresponding with the dominance of the ST11-KL64 CR-HvKp clone in that region [7, 8]. This lineage has emerged through the horizontal transfer of virulence plasmids to pre-existing carbapenem-resistant *K. pneumoniae* strains. Consequently, the pooled estimates may largely represent outcomes associated with this highly successful clone, limiting generalizability to other regions where distinct lineages such as ST23 predominate. These findings reinforce the importance of global genomic surveillance to monitor the evolution and spread of convergent CR-HvKp lineages.

### Study limitations

This meta-analysis has several limitations. Substantial statistical heterogeneity (I^2^ > 88%) was observed for all major outcomes, largely driven by the lack of standardized diagnostic definitions. The predominance of studies from China introduces geographical bias, and most included studies were retrospective, increasing the risk of selection and reporting bias. For the analysis of microbiological failure one study did not mention the timeline of bacterial clearance [52] and one study the HvKp isolates were carbapenem resistant strains [85]. Although publication bias appeared minimal, small studies with extreme findings may still be overrepresented. Despite these constraints, sensitivity analyses confirmed that no single study significantly altered the pooled estimates, supporting the robustness of the overall conclusions.

### Future outlook and recommendations

Despite these limitations, our findings have significant implications. Clinicians must maintain a high index of suspicion for HvKp in patients with invasive *K. pneumoniae* syndromes. For future research, the most urgent priority is the establishment of a global consensus definition for HvKp. The literature shows the limitations of single criteria like the string test and the impracticality of animal models for routine diagnostics. Molecular markers, particularly panels including genes for siderophore systems (*iucA*, *iroB*), capsule regulation (*rmpA*, *rmpA2*), and novel markers like *peg-344*, have shown high diagnostic accuracy.

Therefore, we recommend the formation of an international working group, convened by public health bodies like the WHO or professional societies like IDSA and ESCMID, to develop a standardized, multi-tiered diagnostic framework. Such a framework could be structured as:

- **Tier 1 (Clinical Suspicion):** Based on a characteristic syndrome (e.g., community-acquired invasive infection, particularly pyogenic liver abscess, in a host without underlying hepatobiliary disease).
- **Tier 2 (Probable HvKp):** Confirmation via a validated multiplex PCR panel targeting a consensus set of key virulence genes (e.g., *iucA*, *rmpA/rmpA2*, *iroB*, and *peg-344*).
- **Tier 3 (Definitive HvKp/Research Standard):** For epidemiological and research purposes, incorporating quantitative virulence assays, such as a standardized siderophore production assay, which has shown high promise for accurately differentiating HvKp from cKp.

Harmonizing diagnostics in this manner would improve the comparability of clinical studies, enable more accurate risk stratification, and strengthen global surveillance. Furthermore, enhanced global genomic surveillance is imperative to monitor the evolution and dissemination of convergent CR-HvKp clones.

## Conclusion

This systematic review and meta-analysis provides a comprehensive synthesis of the clinical outcomes associated with HvKp infections and highlights how the absence of a standardized definition contributes to significant heterogeneity in reported estimates. The pooled data confirm HvKp as a major cause of invasive disease, frequently presenting with liver abscess, metastatic spread, and septic shock.

Importantly, the emergence of CR-HvKp appears to substantially worsen clinical outcomes, with pooled mortality exceeding 50% and more than 12-fold higher odds of death compared to carbapenem-susceptible strains. These findings suggest that adverse outcomes are driven by the combined effects of virulence and resistance rather than virulence alone.

While these results underscore a growing clinical concern, they should be interpreted with caution given the heterogeneity of definitions, regional concentration of studies, and predominance of retrospective designs. A globally harmonized diagnostic framework and expanded genomic surveillance are essential next steps to better characterize and contain this evolving threat.

## Supplementary Information

Below is the link to the electronic supplementary material.

Supplementary file1 (PDF 146 KB)

Supplementary file2 (PDF 289 KB)

## Data Availability

Data supporting the findings of this study are available from the corresponding author upon reasonable request.
