# Supplementary material for: Definitional ambiguity and the dual threat of Hypervirulent Klebsiella pneumoniae infections: a systematic review and meta-analysis
Source: Infection. 2025 Dec 10;54(2):645–81. doi: 10.1007/s15010-025-02708-4 (PMC13021745; doi:10.1007/s15010-025-02708-4)
Supplement: Supplementary file 1 — Supplementary file1 (PDF 146 KB) [file 15010_2025_2708_MOESM1_ESM.pdf]

Supplementary table-1 Critical appraisal of literature

Author ID	Q1: Are inclusion criteria clearly defined in the study?	Q2: Are the subjects and settings clearly described?	Q3: Was the exposure measured in a valid and reliable way?	Q4: Was the condition measured in a standard, reliable way for all participants?	Q5: Was appropriate statistical analysis performed for the study objectives?
Zhou et al; 2024	Yes	Yes	Yes	Yes	Yes
Huang et al; 2022	Yes	Yes	Yes	Yes	Yes
Kamau et al; 2022	Yes	Yes	Yes	Yes	N/A
Jung et al; 2013	Yes	Yes	Yes	Yes	Yes
Li et al; 2014	Yes	Yes	Yes	Yes	Yes
Liu et al; 2014	Yes	Yes	Yes	Yes	Yes
Qu et al; 2015	Yes	Yes	Yes	Yes	Yes
Yan et al; 2016	Yes	Yes	Yes	Yes	Yes
Yu et al; 2016	Yes	Yes	Yes	Yes	Yes
Zhang et al; 2016	Yes	Yes	Yes	Yes	Yes
Wu et al; 2017	Yes	Yes	Yes	Yes	Yes
Guo et al; 2017	Yes	Yes	Yes	Yes	Yes
Li et al; 2018	Yes	Yes	Yes	Yes	Yes
Xu et al; 2018	Yes	Yes	Yes	Yes	Yes
Liu et al; 2018	Yes	Yes	Yes	Yes	Yes
Liu and Guo; 2018	Yes	Yes	Yes	Yes	Yes
EL-Mahdy et al; 2018	Yes	Yes	Yes	Yes	Yes
Liu and Guo; 2019	Yes	Yes	Yes	Yes	Yes
Xu et al; 2019	Yes	Yes	Yes	Yes	Yes
Namikawa et al; 2019	Yes	Yes	Yes	Yes	Yes
Harada et al; 2019	Yes	Yes	Yes	Yes	Yes
Zhao et al; 2020	Yes	Yes	Yes	Yes	Yes
Hwang et al; 2020	Yes	Yes	Yes	Yes	Yes
Liu et al; 2020	Yes	Yes	Yes	Yes	Yes
Su et al; 2021	Yes	Yes	Yes	Yes	Yes

Ding et al; 2022	Yes	Yes	Yes	Yes	Yes
Yang et al; 2022	Yes	Yes	Yes	Yes	Yes
Sheng et al; 2022	Yes	Yes	Yes	Yes	Yes
Vandhana et al; 2022	Yes	Yes	Yes	Yes	Yes
Raj et al; 2022	Yes	Yes	Yes	Yes	Yes
Huang et al; 2023	Yes	Yes	Yes	Yes	Yes
Kim et al; 2023	Yes	Yes	Yes	Yes	Yes
Yang et al; 2023	Yes	Yes	Yes	Yes	Yes
Rafat et al; 2018	Yes	Yes	Yes	Yes	Yes
Cubero et al; 2016	Yes	Yes	Yes	Yes	Yes
Guo et al; 2016	Yes	Yes	Yes	Yes	Yes
Hao et al; 2019	Yes	Yes	Yes	Yes	Yes
Chen et al; 2022	Yes	Yes	Yes	Yes	Yes
Yang et al; 2020	Yes	Yes	Yes	Yes	Yes
Lee et al; 2006	Yes	Yes	Yes	Yes	Yes
Yu et al; 2006	Yes	Yes	Yes	Yes	Yes
Peirano et al; 2013	Yes	Yes	Yes	Yes	Yes
Fauvet et al; 2020	Yes	Yes	Yes	Yes	Yes
Zhuo et al; 2025	Yes	Yes	Yes	Yes	Yes
Tang et al; 2025	Yes	Yes	Yes	Yes	Yes
Liu et al; 2025	Yes	Yes	Yes	Yes	Yes
Chen et al; 2025	Yes	Yes	Yes	Yes	Yes
Sun et al; 2025	Yes	Yes	Yes	Yes	Yes
Nannini et al; 2024	Yes	Yes	Yes	Yes	Yes
Moutel et al; 2024	Yes	Yes	Yes	Yes	Yes
Hyun et al; 2024	Yes	Yes	Yes	Yes	Yes
Guo et al; 2023	Yes	Yes	Yes	Yes	Yes
Khairuddin et al; 2023	Yes	Yes	Yes	Yes	Yes
Jin et al; 2023	Yes	Yes	Yes	Yes	Yes
Yadav et al; 2023	Yes	Yes	Yes	Yes	Yes
Li et al; 2021	Yes	Yes	Yes	Yes	Yes

Togawa et al; 2020	Yes	Yes	Yes	Yes	Yes
Lin et al; 2020	Yes	Yes	Yes	Yes	Yes
Kim et al; 2020	Yes	Yes	Yes	Yes	Yes
Tang et al; 2020	Yes	Yes	Yes	Yes	Yes
Li et al; 2020	Yes	Yes	Yes	Yes	Yes
Zhou et al; 2021	Yes	Yes	Yes	Yes	Yes
Hyun et al; 2019	Yes	Yes	Yes	Yes	Yes
Chen et al; 2018	Yes	Yes	Yes	Yes	Yes
Zhan et al; 2017	Yes	Yes	Yes	Yes	Yes
Xiao et al; 2017	Yes	Yes	Yes	Yes	Yes
Ye et al; 2016	Yes	Yes	Yes	Yes	Yes
Zhang et al; 2019	Yes	Yes	Yes	Yes	Yes
Zhao et al; 2019	Yes	Yes	Yes	Yes	Yes
Lin et al; 2018	Yes	Yes	Yes	Yes	Yes
Kim et al; 2018	Yes	Yes	Yes	Yes	Yes
Candra et al; 2023	Yes	Yes	Yes	Yes	Yes
Yang et al	Yes	Yes	Yes	Yes	Yes
Zhang et al	Yes	Yes	Yes	Yes	Yes
Wei et al	Yes	Yes	Yes	Yes	Yes
Shankar C et al	Yes	Yes	Yes	Yes	Yes
Ouyang et al	Yes	Yes	Yes	Yes	Yes
Pan H et al	Yes	Yes	Yes	Yes	Yes
