# Supplementary material for: Definitional ambiguity and the dual threat of Hypervirulent Klebsiella pneumoniae infections: a systematic review and meta-analysis
Source: Infection. 2025 Dec 10;54(2):645–81. doi: 10.1007/s15010-025-02708-4 (PMC13021745; doi:10.1007/s15010-025-02708-4)
Supplement: Supplementary file 2 — Supplementary file2 (PDF 289 KB) [file 15010_2025_2708_MOESM2_ESM.pdf]

**Supplementary Table-2. Resistance to old (conventional) and new β-lactam/β-lactamase inhibitor (BLBLI) combinations and rate of ESBL- and KPC-producing hypervirulent Klebsiella pneumoniae isolates, rate of microbiological failure among HvKp infected patients**

| Study (Year) | Region | HvKp phenotype/genotype | ESBL-producing (%) | KPC-producing (%) | Resistance to OLD BLBLI combinations (%) | Resistance to NEW BLBLI combinations (%) | Microbiological failure (%) | Notes / Additional Findings |
|---|---|---|---|---|---|---|---|---|
| Jung (2013) [29] | Korea | Hypermucoviscous phenotype (String test >5 mm) | 0% (0/14) | 0% (0/14) | Piperacillin-Tazobactam: 0% (0/14) (Susceptible) | NR | NR | ST23 was the dominant hvKP clone. Authors hypothesize a fitness cost or plasmid incompatibility prevents ESBL acquisition in hvKP. |
| Li (2014) [2] | China | Hypermucoviscous phenotype (String test >5 mm) | 17% (5/29) | 0% (0/29) | Ampicillin-Sulbactam: 34% (10/29); Amoxicillin-Clavulanate: 21% (6/29); Piperacillin-Tazobactam: 14% (4/29) | NR | NR | Resistance in hvKP was observed to be increasing over the study period. KPC was found only in cKP isolates. |
| Liu (2014) [33] | China | Hypermucoviscous phenotype (String test >5 mm) | 9.1% (2/22) | 0% (0/22) | Ampicillin-Sulbactam: 27.3% (6/22); Amoxicillin-Clavulanic acid: 9.1% (2/22); Ticarcillin-Clavulanate: 9.1% (2/22); Piperacillin-Tazobactam: 4.5% (1/22) | NR | NR | ST23 was the dominant hvKP clone. One KPC-producer was found, but it was a cKp strain. |

| | | | | | | | | |
|---|---|---|---|---|---|---|---|---|
| Qu (2015) [12] | China | Hypermucoviscous phenotype (String test >5 mm) | 7.7% (1/13) | 0% (0/13) | Cefoperazone-Sulbactam: 0% (0/13) (Susceptible); Piperacillin-Tazobactam: 2.2% (1/45 total isolates, specific hvKP data NR); Ampicillin-Sulbactam: 6.5% (specific hvKP data NR) | NR | NR | Isolates from liver abscess |
| Yan (2016) [13] | China | Genotype (p-rmpA+, iroB+, iucA+) | 7.1% (1/14) | 0% (0/14) | Ampicillin-Sulbactam: 7.1% (1/14); Piperacillin-Tazobactam: 0% (0/14) (Susceptible) | NR | NR | ST23 was the dominant hvKP clone and showed evidence of clonal spread. One ESBL-hvKP was ST29/K54 |
| Yu (2016) [24] | Taiwan | Hypermucoviscous phenotype, rmpA, or rmpA2 gene | 100% (19/19) | NR | NR | NR | NR | All isolates in this study were ESBL-producers by design. Hypervirulence did not affect mortality in this specific cohort. |
| Zhang (2016) [14] | China | Aerobactin gene detection | 12.6% (11/87) | NR | Piperacillin-Tazobactam: 1.1% | NR | Infection Relapse: 2/71 (2.8%);<br><br>Persistent infection after 72-h treatment: 7/77 (9.1%) | Most ESBL-hvKP isolates carried blaCTX-M genes. The study notes the emergence of carbapenem-resistant hvKP in clinical settings. |
| Wu (2017) [25] | China | Positive string test AND/OR rmpA AND/OR | 17.9% (5/28) | 3.6% (1/28) | Piperacillin-Tazobactam: 28.6% | NR | NR | A KPC-2-positive hvKP (ST23) was isolated from a department |

| | | | | | | | | |
|---|---|---|---|---|---|---|---|---|
| | | aerobactin gene (≥2 of 3 criteria) | | | | | | experiencing an outbreak of KPC-2-positive classic K. pneumoniae (ST11). |
| Guo (2017) [11] | China | Hypermucoviscous phenotype (String test >5 mm) | NR | NR | Ampicillin-Sulbactam: 9.5%; Piperacillin-Tazobactam: 4.8% | NR | NR | Four carbapenem-resistant HMKP isolates were identified. K2 was the most common serotype, and hypertension was a newly identified risk factor for infection |
| Li (2018) [34] | China | Genotype (p-rmpA+ AND iucA+) | NR | 57.1% (20/35) | Ampicillin-sulbactam: 62.9%; Piperacillin-tazobactam: 57.1% | NR | NR | A high prevalence of KPC in hvKP was observed. ST11, a clone typically associated with classic K. pneumoniae, was the dominant clone among hvKP isolates (48.6%). |
| Xu (2018) [35] | China | Hypermucoviscous (HM) phenotype (string test) | NR | 4.3% (3/69) of HMKP isolates carried blaKPC. | NR | NR | NR | The 3 KPC+/HM+ isolates belonged to diverse STs (ST11, ST15, ST893). 14-day mortality for KPC+/HM+ BSI was 100% (3/3). |
| Liu (2018) [36] | Chine | Aerobactin positive (genotype | 26.0% (25/96) | NR, but notes 11 CR-hvKp isolates (carbapenem-resistant phenotype). | Ampicillin-Sulbactam: 20.0% (19/96); Piperacillin- | NR | NR | ST23 was the predominant clone. Surgery within 1 month |

| | | | | | | | | |
|---|---|---|---|---|---|---|---|---|
| | | | | | Tazobactam: 3.8% (4/96) | | | was an independent risk factor for CR-hvKp infection. |
| El-Mahdy (2018) [38] | Egypt | hvKP defined by iucA or iroB gene positivity. | 25% (1/4) | NR | Amoxicillin-Clavulanate: 75% (3/4); Cefoperazone-Sulbactam: 75% (3/4) | NR | NR | All 4 hvKP isolates were MDR. K1 and K2 serotypes were present. rmpA genes were significantly associated with hvKP. |
| Liu (2019) [32] | China | Hypermucoviscous (phenotype) AND Aerobactin positive (genotype) | 16.3% (13/80) | NR, but notes 2 CR-hvKp isolates. | NR, but Ampicillin/Sulbactam and Piperacillin/Tazobactam were tested. | NR | Relapse: 6.3% (5/80) | 20% of hvKp were MDR. Infection in ICU and indwelling stomach tubes were independent risk factors for ESBL-HvKp. |
| Xu (2019) [39] | China | pLVPK-like virulence plasmid | NR, but $bla_{CTX\text{-}M\text{-}9}$ group detected in 59.1% (13/32) of HvKp strains | 68.2% (15/22 hvKp strains were $bla_{KPC\text{-}2}$ producers) | Ampicillin-Sulbactam: 77.3% (Total Kp isolates); Piperacillin-Tazobactam: 68.2% (Total Kp isolates) | NR | NR | KPC-2/pLVPK plasmid co-occurrence confirmed. Predominant clones: ST11 (80%), CC23 (13.3%). |
| Yang (2022) [83] | China | Genotype: Presence of iucA, iroB, peg-344, rmpA, or rmpA2. | >50% ($bla_{CTX\text{-}M\text{-}65}$ and $bla_{SHV\text{-}11}$ ) | 100% ($bla_{KPC\text{-}2}$) | Piperacillin-Tazobactam: 100%; Cefoperazone-Sulbactam: 100%<br><br>Specific HvKp data is NR | NR | NR | ST11 Kp infection was an independent risk factor for 30-day mortality (38.3%) and elevated SOFA scores. All ST11 isolates were |

| | | | | | | | | |
|---|---|---|---|---|---|---|---|---|
| | | | | | | | | multidrug-resistant (MDR). |
| Zhang (2022) [84] | China | Genotype: All ST11-KL64 strains carried virulence genes (rmpA2, iucABCD, iutA). | NR | 87.0% of all CRKP (127/146) carried $bla_{KPC-2}$.<br><br>Specific HvKp data is NR | NR | NR | NR | ST11-KL64 is hypothesized to have evolved from ST11-KL47 by acquiring virulence plasmids. |
| Wei (2022) [85] | China | Genotype: Presence of rmpA, rmpA2, iucA, iroN, or peg-344. | NR | 98.8% of all CRKP (79/80) carried $bla_{KPC-2}$.<br><br>Cr-hvKp=51<br>CR-non-hvKp=29 | Piperacillin-Tazobactam: 100%; Cefoperazone-Sulbactam: 100% | Ceftazidime-Avibactam: 2.0%; Imipenem-Avibactam: 3.9% | Persistent infection after 72h treatment: 78.4% (40/51).<br><br>Bacterial clearance after 72hrs was seen only in 3.9% of CR-HvKp group | ST11-K64 was an independent predictor for CR-hvKP infection in older ICU patients. |
| Shankar (2018) [86] | India | Phenotype: String test positive. | NR | 22.2% ($bla_{KPC}$); 40.7% ($bla_{NDM}$); 37.0% ($bla_{NDM}$ +$bla_{OXA-48-like}$) | NR | NR | NR | Extremely high 30-day mortality (74.1%) in patients with string test positive CRKP bacteremia. |
| Ouyang (2022) [87] | China | Genotype: Presence of iucA, iroN, rmpA, or prmpA2. | 39.0% ($bla_{CTX-M-65}$) | 95.1% ($bla_{KPC-2}$) | Piperacillin/Tazobactam: 100% | NR | NR | ST11 KPC-2-producing CR-hvKP was the dominant clone, with evidence of clonal transmission. |
| Pan (2019) [88] | China | Phenotype: String test positive. | NR | 100% of all CPKP (66/66) carried $bla_{KPC-2}$. | Amoxicillin-clavulanic acid: 100% (66/66); Cefoperazone-Sulbactam: 97% (64/66) | NR | NR | ST11 was the dominant clone in hvCPKP isolates (86.7%). |

| | | | | | | | | |
|---|---|---|---|---|---|---|---|---|
| Hwang (2020) [42] | Korea | Positivity for both rmpA and iutA virulence genes | 25.6% (10/39 hvKp strains) | 0% (Meropenem susceptibility: 100%) | Piperacillin-Tazobactam: 25.6% (10/39 hvKp). Ampicillin-Sulbactam: 28.2% (11/39 hvKp). | NR | NR | HvKP strains had significantly higher prevalence of K1 serotype (35.9%). |
| Harada (2019) [43] | Japan (23 hospitals) | Carriage of any of the virulence genes:rmpA,rmpA 2, iroBCDN, iucABCD, and iutA | 3.8% (1/26 hvKp isolate carried $bla_{CTX\text{-}M\text{-}2}$) | 7.7% (2/26 hvKp isolates carried carbapenemase genes: $bla_{IMP\text{-}6\ or}$ $bla_{GES\text{-}4}$) | NR | NR | NR | HvKp associated with disseminated infections (Risk Ratio, 6.58). |
| Zhao (2020) [41] | China | At least two indicators: positive string test, gene-positive, and/or aerobactin-positive status | 15.4% (4/26 hvKp strains) | 7.7% (2/26 hvKp strains were carbapenem-resistant) | NR | NR | NR | HvKp prevalent in Surgical Site Infections (51.0% overall). |
| Namikawa (2019) [40] | Japan | Hypermucoviscous phenotype (HMV) based on positive string test (>5mm viscous string) | 0% (0/24 hvKp strains) | NR | NR | NR | NR | Abscess and no antibiotic exposure were independent predictors of hvKP bacteremia. |
| C Liu (2018) [37] | China | Aerobactin-positive, hypermucoviscosit y phenotype, K1, rmpA, rmpA2, magA clustered | 38.2% of HvKp isolates were ESBL-producing | 8 CR-hvKp (specific KPC gene NR) | Ampicillin-Sulbactam 38.2%, Piperacillin-Tazobactam 23.5% | NR | NR | ST23, ST37, ST2906 more likely to induce lethal VAP; coexistence of MDR and ESBL traits in some HvKp. |
| Liu (2020) [44] | China | Genotype: prmpA, prmpA2, iucA, iroB, peg-344 | 47.2% in nosocomial , 26.3% in healthcare-associated isolates | 26.4% in nosocomial, 10.5% in healthcare-associated isolates | Piperacillin–Tazobactam: 28.3% resistant; Ampicillin–Sulbactam: 37.7%; Amoxicillin–Clavulanate: 31.1%; Cefoperazone–Sulbactam: 22.6% | NR | NR | 91% of HvKp infections were nosocomial or healthcare-associated. MDR-HvKp mostly ST11; ESBL genes frequently coexisted with |

| | | | | | | | | |
|---|---|---|---|---|---|---|---|---|
| | | | | | | | | virulence plasmids. |
| Su (2021) [45] | China | Defined by rmpA, rmpA2, and positive string test | NR | 17.6% (12/68 HvKp); all except 2 harbored KPC | Piperacillin-Tazobactam 17.6%, Cefoperazone-Sulbactam 19.1% | NR | NR | All HvKP sensitive to tigecycline/colistin; most KPC genes were KPC-2. Coexistence of high virulence and resistance rare, but present. Carbapenemase-producers mostly ST11. |
| Ding 2021 [46] | China | Aerobactin+ & Galleria model+ | 7.5% (4/53) | 1.9% (1/53, blaKPC-2+, SHV-11+) | Piperacillin-Tazobactam 3.8%, CAZ-CLA 1.9%, CTX-CLA 3.8% | NR | NR | 53/123 isolates HvKP. ST23/K1 dominant (21/53, 39.6%). High resistance in non-HvKP; MDR-HvKP 15.1%. |
| Yang 2022 [30] | China | peg-344 or iucA+ | blaSHV in all, blaSHV-190 was the most prevalent 52.2% | 1/69 hvKP resistant to meropenem/ertapenem, no carbapenemase gene | Piperacillin/tazobactam, Cefoperazone/sulbactam were less than 10% | NR | NR | K1/ST23 majority. All strains with oqxA, oqxB, fosA. mceG only in K1. Various new STs detected. |
| Sheng 2022 [31] | China | Aerobactin + | NR | 0 (None) | Ampicillin-Sulbactam-3.4% | NR | NR | BSI cases; K1/K2 predominant, ST23 prevalent, multiclonal origin, rmpA 89.7%, iroBCD 86.2%, ybtS 72.4%. |
| Vandhana (2022) [3] | India | Aerobactin gene positive | 44.4% (8/18) | 16.7% (3/18) Carbapenem-resistant | Piperacillin-tazobactam: 29% (overall Kp); | NR | NR | High mortality rate in HvKp infections (87.5%) compared |

| | | | | | | | | |
|---|---|---|---|---|---|---|---|---|
| | | | | | Cefoperazone-sulbactam: 32% (overall Kp) | | | to cKp (35.7%). 44.4% of HvKp isolates were multidrug-resistant (MDR). |
| Raj (2022) [47] | India | Aerobactin gene positive | 78.5% (11/14) carried bla_{CTX-M-15} | 35.7% (5/14) carried bla_{NDM}; 71.4% (10/14) carried bla_{OXA} | 91.6% (11/12) in hospital-acquired HvKp | NR | NR | Study differentiated between CA and HA-HvKp. High resistance rates were noted in HA-HvKp. ST2096 was the most common clone. |
| Huang (2023) [7] | China | rmpA, rmpA2, iucA, iroB, or peg-344 positive | NR (All 8 NDM-1-KPC-2 strains carried bla_{SHV} and bla_{TEM}) | 53.3% (24/45) Carbapenemase producers; 17.8% (8/45) co-produced NDM-1 and KPC-2 | NR | Ceftazidime-avibactam: 100% (8/8) resistance in NDM-1-KPC-2 co-producers | NR | All 8 NDM-1-KPC-2 co-producing strains belonged to ST11-K64. These strains were associated with high mortality (6/8 patients died). |
| Kim (2023) [27] | Republic of Korea | String test >5 mm (hmvKp) | NR (Cefotaxime resistance: 11.9%) | NR | NR | NR | NR | Study focused on non-hepatobiliary bacteremia. hmvKp was associated with purulent/necrotizing infections (31.3%) and metastatic infections (9.0%) |
| Yang (2023) [48] | China | ≥4 of 5 genes (iroB, iucA, rmpA, rmpA2, peg-344) | NR | NR | Cefoperazone-sulbactam: 15.2% (5/33); Piperacillin-tazobactam: 15.2% (5/33) | NR | Antibiotic tolerance: HvKp isolates were "not easily killed" | ST23-K1 was the dominant HvKp clone. ST11 strains (both HvKp and cKp) were MDR. |

| | | | | | | | | |
|---|---|---|---|---|---|---|---|---|
| | | | | | | | and were "more tolerant to antibiotics" than cKp isolates in time-kill assays. | WBC/PCT and CRP/PCT ratios were identified as potential clinical markers. |
| Rafat (2018) [49] | France | Hypermucoviscosity (string test) and virulence gene analysis (rmpA, iutA, etc.) | 0% | NR | All isolates displayed "wild-type" susceptibility (resistance restricted to amoxicillin/ticarcillin). | NR | NR | Predominant clones were ST23 (K1) and ST86 (K2). Associated with higher rates of multi-organ failure (83.3%). |
| Cubero (2016) [50] | Spain (Barcelona) | Hypermucoviscosity (string test) and presence of magA and/or rmpA genes. | NR (but isolates were described as "fully susceptible") | NR | All magA(+) and/or rmpA(+) isolates were "fully susceptible" to all antimicrobials tested except ampicillin. | NR | Two patients had recurrent bacteremia with the same strain, including one ST23-K1 case with relapse as a liver abscess. | Hypervirulent clones (CC23K1, CC86K2, CC65K2, CC380K2) were infrequent (2.7% of all bacteremia isolates). String test alone was deemed not fully reliable. |
| Guo (2016) [51] | China | Hypermucoviscosity phenotype (string >5 mm). | 14.3% (2/14) | NR | NR | NR | NR | HvKp-VAP associated with higher bacteremia (35.7%) and mortality (57.1%). ST23, ST86, and ST65 identified among HvKp isolates. |
| Hao (2019) [52] | China | Hypermucoviscosity (string test >5 mm). | 6.1% (2/33) | 0% | Piperacillin/tazobactam: 0% | NR | 45.5% (calculated as 100% - 54.5% | ST23 was the major epidemic strain (39.4% of HvKp). K1 and K2 |

| | | | | | | | | |
|---|---|---|---|---|---|---|---|---|
| | | | | | | | bacterial clearance rate). | were the most common serotypes. |
| Chen (2022) [53] | China | Hypermucoviscosity phenotype (string test >5 mm). | NR | NR | NR | NR | NR | HvKp infection associated with a higher incidence of septic shock (16.7%). |
| Yang (2020) [54] | China | PCR for virulence genes (p-rmpA, p-rmpA2, iucA, iroB, peg-344, peg-589) | 1.7% (1/59) | NR | Ampicillin-Sulbactam: 1.7% (1/59) | NR | NR | HvKp isolates were significantly more susceptible to most antimicrobials compared to cKp. Main capsular serotypes were K2, K57, and K1. |
| Lee (2006) [55] | Taiwan | Hypermucoviscosity (string test >10 mm) and magA gene detection | NR | NR | NR | NR | NR | Study focused on the association between the HV phenotype and invasive clinical syndromes |
| Peirano (2013) [56] | Canada (Calgary) | Hypermucoviscosity (string test ≥5 mm) and detection of rmpA, magA, K1, K2, K5 | NR | NR | NR | NR | NR | 8.2% of community-acquired bacteremia isolates were hypermucoviscous. Associated with rmpA and K2 serotype. Liver abscess was the most common presentation. No resistance data reported. |

| | | | | | | | | |
|---|---|---|---|---|---|---|---|---|
| Fauvet (2020) [57] | New Caledonia | Hypermucoviscous (string test >5 mm) and molecular analysis for K1, K2, K5 serotypes | 6.7% (1/15) | NR | NR | NR | Persistence of positive blood cultures after 3 days of adequate therapy: 26.7% (4/15) | HvKp accounted for 27% of all KP bacteremia. Associated with community-acquired infections. The K1/K2 genotype was present in 86.6% of hypermucoviscous strains |
| Zhuo (2025) [16] | China | Mouse lethality model; operationally defined as K1, K2, K20 serotypes | 1.4% (1/70) had blaCTX-M-15 | 0% (0/70) had blaKPC-2 | NR | NR | NR | HvKp had better clinical outcomes (lower mortality) than cKp. HvKp isolates were significantly more susceptible to antibiotics than cKp. |
| Tang (2025) [58] | China | Genotype: Co-presence of iucA, iroB, peg-344, rmpA, and rmpA2. | 1.6% (2/127) | 0% | NR | NR | Initial treatment failure: 20.6% (26/126); Re-infection: 4.8% (6/126) | This cohort represents a largely susceptible HvKp population. The predominant clone was ST23. Despite high susceptibility, clinical outcomes were severe. |
| Liu (2025) [8] | China | Genotype: Presence of rmpA, rmpA2, iroB, iucA. | NR | NR, but blaKPC-2 was the most prevalent carbapenemase gene. | NR | NR | NR | Predominant clone was ST11-KL64 hv-CRKP. Showed significant nosocomial transmission, especially in the ICU. |

| | | | | | | | | |
|---|---|---|---|---|---|---|---|---|
| Chen (2025) [59] | China | Genotype: Presence of at least one of rmpA, rmpA2, iroB, iucA, or peg-344. | NR | 61.8% (42/68) of all CRKP carried blaKPC-2. Prevalence in CR-HvKP subgroup was significantly higher (91.7%) than in CR-non-HvKP (28.1%). | NR | NR | NR | ST11-KL64 was the dominant clone. All 36 CR-HvKP isolates harbored iucA. Only 22.2% of CR-HvKP were string test positive. |
| Sun (2025) [60] | China | Phenotype: String test mm. | 6.5% (15/232) of hmKp patients had MDR/ESBL isolates on admission. | NR | Piperacillin-Tazobactam: 1% Resistance was significantly lower in hmKp compared to n-hmKp. Cefoperazone-Sulbactam: 2%. | NR | NR | hmKp isolates were more susceptible to older BLBLIs than n-hmKp isolates. |
| Yu (2006) [23] | Taiwan | Phenotype: String test mm. Genotype: Presence of rmpA and/or magA. | NR | NR | NR | NR | NR | Found a strong association between rmpA and abscess formation. No antimicrobial susceptibility data were reported. |
| Nannini (2024) [61] | Argentina | Hypermucoviscous phenotype (positive string test) & virulence genes (rmpA, iroB, iucA) | NR | NR | 0% | NR | NR | Study on cryptogenic liver abscesses (CLA). All isolates were community-acquired. Two patients required eye enucleation due to endophthalmitis. Clones included ST23, ST65, ST86. |
| Moutel (2024) [62] | France | Genotypic: rmpA and iutA positive | NR | NR | Acquired beta-lactam resistance: 11% (1/9) | NR | NR | Study of bacteremia in an |

| | | | | | | | | |
|---|---|---|---|---|---|---|---|---|
| | | | | | (beta-lactamase inhibitor-resistant penicillinase) | | | ICU. 13% (9/70) of K. pneumoniae BSI were HvKp. |
| Hyun (2024) [63] | South Korea | Clinical syndrome: Community-acquired liver abscess (CLA) (n=34) | 2.9% (1/34) | NR | Amoxicillin-Clavulanate: 2.9% (1/34); Piperacillin-Tazobactam: 5.9% (2/34) | NR | NR | rmpA was the most significant risk factor for CLA. CLA group had significantly higher antibiotic susceptibility overall compared to non-CLA group. |
| Huang (2023) [64] | China | Phenotypic (positive string test) AND Genotypic (one or more of rmpA, rmpA2, iucA, iroB, magA, peg344) | NR | NR | NR | NR | NR | Study of 116 patients to develop prognostic models. |
| Guo (2023) [9] | China | Hypermucoviscous phenotype (positive string test) & genomic analysis | NR | NR | PLA group: Cefoperazone-Sulbactam: 44% (11/25); Piperacillin-Tazobactam: 24% (6/25)<br>VAP group: Cefoperazone-Sulbactam: 71.43% (10/14); Piperacillin-Tazobactam: 57.14% (9/14) | NR | NR | Comparison study. PLA isolates had lower resistance but higher virulence scores. VAP isolates had significantly higher resistance scores. |
| Khairuddin (2023) [65] | Malaysia | Positive string test AND K1 or K2 serotype gene | NR | NR | NR | NR | NR | Pneumonia was the leading diagnosis (70.5%). Mortality rate was 12%. All hvKp isolates were positive for rmpA, |

| | | | | | | | | |
|---|---|---|---|---|---|---|---|---|
| | | | | | | | | rmpA2, iucA, and peg-344. |
| Jin (2023) [26] | China | Hypermucoviscous (HmKp) phenotype (positive string test, mm) | HmKp carried more SHV-type ESBL genes than non-HmKp | NR | NR | NR | NR | Diabetes mellitus and liver abscess were significant risk factors. Dominant STs in HmKp were ST23 (27.8%) and ST65 (16.7%). |
| Yadav (2023) [66] | India | Hypermucoviscous phenotype (positive string test, mm) | NR | 0% | Amoxicillin-Clavulanate: 42.4%; Piperacillin-Tazobactam: 36.3% | NR | NR | Carbapenem resistance in hvKP was 30.3% (), driven by OXA-48/OXA-181 (50%) and NDM (20%). Mortality was 30.3%. |
| Li (2021) [67] | China | Aerobactin gene (*iucA*) positive | NR | 30.8% (8/26) | Amoxicillin-Clavulanate: 38.5% (8/26) | NR | NR | All 8 CR-hvKp isolates were ST11, KPC-producing, and non-hypermucoviscous (string test negative). Dominant STs were ST23 (30.8%) and ST11 (30.8%). |
| Togawa (2020) [68] | Japan | Clinical definition: K. pneumoniae BSI with liver abscess formation | 1.8% | 0% | NR | NR | NR | Hypermucoviscosity (50%) and K1 serotype (50%) were significantly associated with liver abscess. |

| | | | | | | | | |
|---|---|---|---|---|---|---|---|---|
| Lin (2020) [17] | Taiwan | Hypermucoviscous phenotype AND rmpA or rmpA2 gene positive | 15.4% ( MDR-HV strains were ESBL-producers: SHV-5, SHV-12) | 0% | Ampicillin-Sulbactam: 15.4%; Piperacillin-Tazobactam: 7.7% | NR | NR | 6.0% () of KPLA episodes were caused by MDR-HV strains. All were "Type I" MDR-HV (classic virulent clones acquiring resistance). Dominant clones were ST23, ST65, ST86. |
| Kim (2020) [69] | Republic of Korea | HvKp defined by positive string test from liver abscess aspirates. | 0% (0/11) in liver isolates. 100% (8/8) in non-K1/K2 stool isolates. | NR | NR | NR | NR | Study compared liver abscess isolates with stool isolates. Liver isolates were ESBL-negative. Stool isolates showed high heterogeneity and resistance. |
| Tang (2020) [28] | China | Positive string test and PCR-amplified *iucA*. | 9.6% (13/135) of isolates were ESBL-producing. | 3.7% (5/135) of isolates were CR-hvKP, all harboring *bla*KPC-2. | Ampicillin/Sulbactam: 17.8% (24/135). Piperacillin/Tazobactam: 3.7% (5/135). Cefoperazone/Sulbactam: 3.0% (4/135). | NR | NR | In-hospital mortality was 11.9%. Admission to ICU and carriage of *iroN* gene were independent prognostic factors for mortality. ST11 was predominant among CR-hvKP. |
| Li (2020) [70] | China | Positive string test and positive for virulence genes (*rmpA*, *rmpA2*, *iroN*, *iucA*). | 7.4% (6/81) | 0% (No carbapenem-resistant HvKp isolated) | Ampicillin/Sulbactam: Resistance rate significantly lower than cKP | NR | NR | Diabetes was an independent risk factor for invasive HvKp. 51.2% of invasive infections were treated with |

| | | | | | | | | |
|---|---|---|---|---|---|---|---|---|
| | | | | | | | | antibiotics plus surgical drainage. ST23 was the predominant ST. |
| Zhou (2021) [71] | China | Hypermucoviscous phenotype (positive string test) and carrying virulence loci (*rmpA2*, *iutA*, *iucA*). All isolates were carbapenem-resistant (CR-hvKP). | All 16 isolates carried *bla*SHV. 68.8% (11/16) carried a *bla*CTX-M gene. | 100% (16/16) carried *bla*KPC-2. | Ampicillin/Sulbactam: 100%. Cefoperazone/Sulbactam: 100%. Piperacillin/Tazobactam: 100%. | NR | NR | Study focused on 16 CR-hvKP isolates. ST11 was the most predominant ST (87.5%). Serotype K64 was most common (81.3%). Mortality was 56.3% (9/16). |
| Hyun (2019) [72] | Korea | Hypermucoviscous phenotype (positive string test). | Community-acquired: 3.2% (3/95). Healthcare-associated: 20% (12/60). | All isolates were susceptible to carbapenems. | Community-acquired: Amoxicillin/Clavulanic acid: 4.2%; Piperacillin/Tazobactam: 3.2%. Healthcare-associated: Amoxicillin/Clavulanic acid: 16.7%; Piperacillin/Tazobactam: 11.7%. | NR | NR | 38% of HvKp was healthcare-associated. Healthcare-associated HvKp had higher antibiotic resistance, were more often non-K1/K2 serotypes, and less often carried *rmpA*. |
| Chen (2018) [73] | China | Hypermucoviscous phenotype (positive string test). HMKP isolates. | ESBLs not detected among carbapenem-susceptible (CS) isolates. | 23.8% (10/42) were CR-HMKP, all producing KPC-2. | Amoxicillin/Clavulanic acid: Resistance reported via MIC90 >32 mg/L for CR-HMKP vs. 4 mg/L for CS-HMKP. | NR | NR | ST11 was unique to CR-HMKP strains. Mortality was significantly higher in patients with CR-HMKP (60.0%) vs. CS-HMKP (6.3%). |
| Zhan (2017) [74] | China | Hypermucoviscous phenotype (positive string test). All isolates were | 71.4% (15/21) harbored *bla*CTX- | 100% (21/21) were positive for *bla*KPC-2. | Ampicillin/Sulbactam: 100%. Piperacillin/Tazobactam: 100%. | NR | NR | An outbreak of ST11 CR-HMKP in an ICU was described. 61.9% |

| | | | | | | | | |
|---|---|---|---|---|---|---|---|---|
| | | carbapenem-resistant (CR-HMKP). | M-65. 95.2% (20/21) harbored *bla*SHV-11. | | | | | of patients had prior carbapenem treatment. Serotype K20 was common (33.3%). |
| Candra (2023) [82] | Indonesia | Positive rmpA and/or iucA genes, positive string test | 0/5 (0%) | 0/5 (0%) | Ampicillin-sulbactam: 0/5 (0%), Piperacillin-tazobactam: 0/5 (0%), Cefoperazone-sulbactam: 0/5 (0%) | NR | NR | hvKp were non-ESBL, non-KPC, 80% with comorbidity, 100% mortality |
| Xiao (2017) [75] | China | Hypermucoviscous phenotype (string test >5 mm) | NR | 8.3% (2/24 carried blaKPC-2) | Ampicillin-Sulbactam: 20.8% Piperacillin-Tazobactam: 8.3% | NR | NR | Carbapenem resistance was found in K2/ST25 and K2/ST65 isolates. |
| Ye (2016) [18] | China | Isolates from pyogenic liver abscess (PLA) | NR | NR | Ampicillin-Sulbactam: 0% Piperacillin-Tazobactam: 0% | NR | NR | All 40 LA-Kp isolates were susceptible to piperacillin-tazobactam and ampicillin-sulbactam. 12.5% of isolates had no plasmid, suggesting chromosomal integration of virulence genes. ST23 was the major sequence type (47.5%). |
| Zhou (2024) [15] | China | Genotypic markers: rmpA, rmpA2, iucA, iroB, peg-344. | NR | 18.8% (3/16 were carbapenem-resistant) | NR | NR | NR | Study on endogenous endophthalmitis (EKPE). Three carbapenem-resistant isolates were avirulent |

| | | | | | | | | |
|---|---|---|---|---|---|---|---|---|
| | | | | | | | | ST11. iroB was the only virulence gene harbored by all isolates. |
| Huang (2022) [76] | China | Genotypic markers: peg-344 and iucA | NR | 41.7% (15/36 carried blaKPC-2) | NR | NR | NR | Study on meningitis cases (2014-2020). Mortality in the Hv-CRKP group was 92.3% (12/13). ST11 was the most common ST (38.9%). |
| Kamau (2022) [77] | USA (Southern California ) | Hypermucoviscous phenotype + genotypic confirmation (≥3 of iucA, iroB, peg-344, rmpA, rmpA2) | 0% | 0% | Piperacillin/tazobactam : 0% | NR | NR | All isolates were pan-drug susceptible. Prevalence was 2.6% of invasive *K. pneumoniae* infections. K1-ST23 was the predominant lineage (10/15). |
| Zhang (2019) [78] | China | Isolates from PLA; characterized by hypermucoviscosit y (30.7%), K1/K2 serotypes, and virulence genes | NR | 3.7% (6/163 were CRKP) | Ampicillin/sulbactam: 10.4% Piperacillin/tazobactam : 1.2% Cefoperazone/sulbacta m: 0% | NR | NR | Large cohort of PLA cases (2016-2017). ST23 was predominant (38.7%). MDR strains were significantly more common in patients with other hepatobiliary diseases (OHD). |
| Zhang (2019) (OHD | | Subgroup of PLA patients with other hepatobiliary diseases (OHD); | NR | 10.3% (3/29 were CRKP) | Ampicillin/sulbactam: 24.1% Piperacillin/tazobactam : 6.9% | NR | NR | This subgroup had significantly higher rates of resistance to |

| | | | | | | | | |
|---|---|---|---|---|---|---|---|---|
| Subgroup) [78] | | | | | | | | multiple antibiotics compared to the non-OHD group. 24.1% of isolates were MDR. |
| Zhang (2019) (non-OHD Subgroup) [78] | | Subgroup of PLA patients without other hepatobiliary diseases | NR | 2.2% (3/134 were CRKP) | Ampicillin/sulbactam: 7.5% Piperacillin/tazobactam: 0% | NR | NR | This subgroup had very low rates of antimicrobial resistance. 3.7% of isolates were MDR. |
| Zhao (2019) [79] | China | Hypermucoviscous phenotype + genotypic markers (e.g., iutA, rmpA) | NR | 96.6% (28/29 carried blaKPC-2) | NR | NR | One patient remained colonized for 115 days despite therapy, with the isolate ultimately causing fatal bacteremia. | Fatal ICU outbreak with 100% mortality. 89.7% (26/29) of isolates were ST11. |
| Lin (2018) [80] | Taiwan | Capsular type K1; n=18 (antimicrobial-resistant group) | NR | NR | NR | NR | NR | Compared 18 resistant K1 isolates to 164 sensitive K1 isolates. 28-day mortality was 50% in the resistant group vs. 10.4% in the sensitive group. Most resistant strains were ST23. |
| Kim (2019) [81] | South Korea | Hypermucoviscous phenotype, K1/K2 serotypes | NR | 1.4% (8/579 carried blaKPC-2) | Ampicillin/sulbactam: 29.5% Piperacillin: 33.9% | NR | NR | Large bloodstream infection (BSI) cohort. Hypermucoviscosi |

| | | | | | | | | |
|---|---|---|---|---|---|---|---|---|
| | | | | | | | | ty was inversely associated with mortality. The pks gene cluster was a risk factor for mortality. |

Aerobactin gene A: iucA; Ampicillin-Sulbactam: AST (antimicrobial susceptibility testing); BSI: bloodstream infection; bla: beta-lactamase gene marker; CA: community-acquired; CAZ-CLA: ceftazidime-clavulanate; CC: clonal complex; Cefoperazone-Sulbactam: AST (antimicrobial susceptibility testing); cKp: classical Klebsiella pneumoniae; CR-hvKp: carbapenem-resistant hypervirulent Klebsiella pneumoniae; CR-KP: carbapenem-resistant Klebsiella pneumoniae; CRPPCT: C-reactive protein to platelet count ratio; CTX-CLA: cefotaxime-clavulanate; CTX-M: cefotaximase-Munich type beta-lactamase; ESBL: extended-spectrum beta-lactamase; HA: hospital-acquired; HMKP: hypermucoviscous Klebsiella pneumoniae; HMV: hypermucoviscosity; HvKp: hypervirulent Klebsiella pneumoniae; ICU: intensive care unit; iroB: salmochelin gene B; K1, K2, K64: capsular serotypes; KPC: Klebsiella pneumoniae carbapenemase; magA: mucoviscosity-associated gene A; MDR: multidrug-resistant; NDM: New Delhi metallo-beta-lactamase; NR: not reported; OHD: other hepatobiliary diseases; OXA: oxacillinase; peg-344: putative transporter gene 344; PLA: pyogenic liver abscess; pLVPK: large virulence plasmid of K. pneumoniae; rmpA: regulator of mucoid phenotype A; rmpA2: regulator of mucoid phenotype A2; SHV: sulfhydryl variable beta-lactamase; SOFA: sequential organ failure assessment; ST: sequence type; TEM: Temoniera beta-lactamase; VAP: ventilator-associated pneumonia; WBCPCT: white blood cell to platelet count ratio
